# Supplementary material for: A transboundary water allocation strategy for the Aral Sea Basin: Integrating the water-food-energy-environment nexus
Source: Innovation (Camb). 2026 Jan 7;7(5):101257. doi: 10.1016/j.xinn.2026.101257 (PMC13147992; doi:10.1016/j.xinn.2026.101257)
Supplement: Document S1. Tables S1–S3 [file mmc1.pdf]

The Innovation, Volume 7

Supplemental Information

A transboundary water allocation strategy for the Aral Sea Basin: Integrating the water-food-energy-environment nexus

Yanan Hu, Guangdong Sun, Weili Duan, Shan Zou, Yanfeng Di, Yaning Chen, Patient Mindje Kayumba, Wei Wei, Philippe De Maeyer, and Peter L.M. Goethals

TEXT S1:

The Random Forest process involves[1]: (1) generating bootstrap samples from the training set (N samples) to construct individual decision trees; (2) randomly selecting m features (m ≪ M) from M input features at each node split and choosing the optimal split point, a key mechanism to reduce overfitting and enhance generalization; and (3) allowing each tree to grow fully without pruning. The final prediction is obtained by averaging the outputs of all trees, balancing individual tree complexity with ensemble generalization error control.

The Random Forest model was assessed using an out-of-sample validation strategy. Specially, 80% of the historical runoff and hydrometeorological data were employed for model calibration, while the remaining 20% were withheld as an independent validation set. This approach is widely recognized in hydrological modelling as a reliable means to evaluate predictive skill and to minimize the risk of overfitting. Model performance, shown in Fig. S1, indicates strong predictive capability, with Coefficient of Determination (R^2) values of 0.72–0.90 and Nash-Sutcliffe efficiency coefficient (NSE) of 0.91–0.96 for the three stations. These results demonstrate that the Random Forest model captures the nonlinear relationships between predictors and runoff and provides robust and reliable out-of-sample predictions.

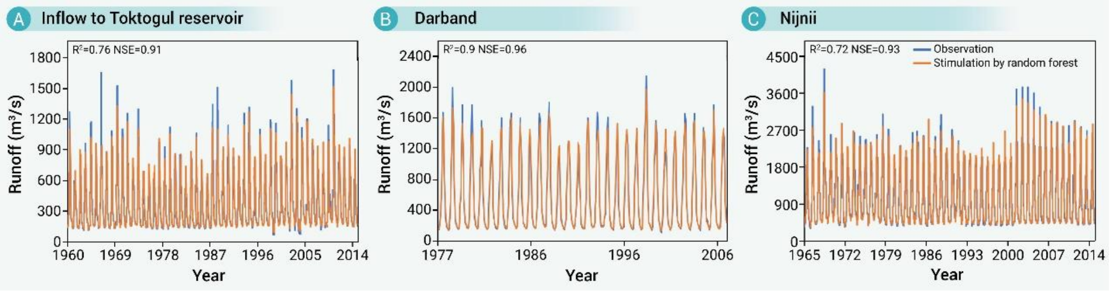

Fig. S1. Comparison and validation of observed and simulated runoff in the Aral Sea Basin

TEXT S2:

The CROPWAT model was used to calculate the irrigation water requirements for five crops. CROPWAT, a tool based on the Penman-Monteith formula, estimates crop water needs and irrigation demand. The equations are as follows:

$$ET_c = K_c * ET_0 \tag{1}$$

Where ET_c is the crop water demand,, K_c is the crop coefficient for different growth stages (parameters for the five Central Asian crops were taken from Liu et al.[2]), and ET_0 the potential evapotranspiration.

$$P_e = P * \frac{4.17 - 0.2 * P}{4.17}, for\ P < 8.3mm/day \tag{2}$$

$$P_e = 4.17 + 0.1 * \mathrm{P}, \mathrm{for\ P} \geq 8.3\mathrm{mm/day} \tag{3}$$

where Pe is effective precipitation and P is the precipitation.

$$CIR = Area * 10 * (ET_0 - P_e)/I \tag{4}$$

where CIR represents the crop irrigation requirement, Area is the crop area, and the constant 10 converts water depth (mm) into water volume (m^3/ha), I is the irrigation efficiency, which in this study considers a combination of flood and drip irrigation with an efficiency of 50%, based on coefficients from Wang et al.[3].

TEXT S3:

To evaluate the robustness of the optimization results under uncertain future conditions, two primary sources of uncertainty were considered: socioeconomic shared pathways and hydrological inflow variability. For each optimization objective, sensitivity was assessed using both mean-based and range-based indicators.[4] Mean-based sensitivity measures the average deviation of objectives under different SSPs and Flow conditions, reflecting the influence of uncertainty on overall trends. Range-based sensitivity captures the difference between maximum and minimum values across scenarios, emphasizing system performance under extreme conditions. Furthermore, a two-way analysis of variance (ANOVA) was applied to partition the outcome variance into contributions from SSP, Flow, and their interaction.[5] The effect size, quantified by η^2 (eta squared), indicates the proportion of variance explained by each factor, thereby identifying the dominant drivers and interaction effects. By integrating mean-based sensitivity, range-based sensitivity, and variance decomposition, this framework provides a comprehensive characterization of how uncertainties propagate into equity, hydropower, agricultural, and environmental objectives, ensuring that comparative evaluations of alternative water allocation strategies remain robust across scenarios.

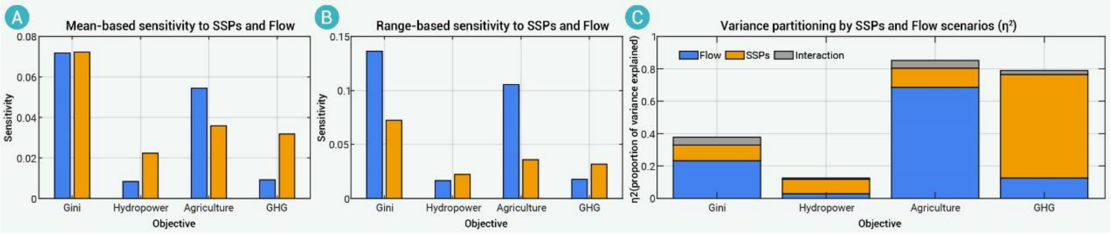

Fig. S2. Sensitivity analysis and ANOVA under SSPs and flow scenarios

Table S1. Details of the CMIP6 global climate models used in this study

Number	Model	Institute	Country
1	CanESM5	Canadian Centre for Climate Modelling and Analysis	Canada
2	CMCC-CM2-SR5	Fondazione Centro Euro-Mediterraneo sui Cambiamenti Climatici	Italy
3	CMCC-ESM2	Fondazione Centro Euro-Mediterraneo sui Cambiamenti Climatici	Italy
4	GFDL-ESM4	NOAA Geophysical Fluid Dynamics Laboratory	USA
5	INM-CM4-8	Institute for Numerical Mathematics	Russia
6	INM-CM5-0	Institute for Numerical Mathematics	Russia
7	IPSL-CM6A-LR	Institute Pierre-Simon Laplace	France
8	MIROC6	Institute of Atmospheric Oceanography, Japan Agency of Marine Earth Science and Technology	Japan
9	NorESM2-LM	Norwegian Climate Centre	Norway
10	NorESM2-MM	Norwegian Climate Centre	Norway
11	TaiESM1	Research Center for Environmental Changes, Academia Sinica	Taiwan, China

Table S2. Nomenclature for parameters and variables.

Indices	Definition
I	Country
J	Reservoir
K	Seasonal pumped hydropower storage reservoir
T	Crop type
X	Crop growing season and non-growing season
PO_i	Population of country i in the basin
W_i	The amount of water that can control in country i (hydropower in upstream countries, agriculture in downstream countries) (m^3)
HW_j	Electricity generation per unit water of reservoir j (kwh/m^3)
WA_{jh}	water available for hydropower of reservoir j (m^3)
HEC_i	Household electricity consumption of country i (kWh)
E_k	Seasonal pumped hydropower storage electricity generation (kWh)
P_{hi}	Household electricity prices in country i (US$/kWh)
P_{bi}	Business electricity prices in country i (US$/kWh)
C_k	total cost of seasonal pumped storage reservoir k (US$)
A	Total crop area in the Aral Sea basin (ha)
A_{it}	Area of crop t in country i (ha)
Y_{it}	Yield per unit area of crop t in country i (ton/ha)
P_{it}	Price of crop t in country i (US$/ton)
P_{it}^{irri}	Irrigation cost per unit area of crop t in country i (US$/ha)
P_{it}^{fert}	Fertilizer cost per unit area of crop t in country i (US$/ha)
N_i	Nitrogen fertilizer application per unit area in country i (kg/ha)
EF_d	Direct emission factor for N fertilizer application
F_{vol}	Fraction of the ammonia volatilization
EF_{vol}	Emission factor of ammonia volatilization
F_{leach}	Fraction of the nitrogen leaching losses
EF_{leach}	Emission factor of nitrogen leaching
A_i^{rice}	Rice harvested area in country i (ha)
EF_{CH_4}	Emission factor of rice
WD_t	Water demand per unit area of crop t (m^3/ha)
WS_e	Ecological water supply for the Aral Sea (m^3)
WD_e	Ecological water demand of the Aral Sea (m^3)
TAW	Total available water (m^3)
WD_{io}	Industrial, municipal and livestock water demand in country i (m^3)
SAW	Seasonal water availability (m^3)
FD	Per capita food demand (ton/per capita)
V	Reservoir water storage (m^3)
V_{min}	Minimum water storage capacity of the reservoir (m^3)
V_{max}	Maximum water storage capacity of the reservoir (m^3)
HD	Hydropower demand in the basin (kWh)

Table S3. The probability of selecting seasonal pumped hydropower storage reservoirs

					SSP2-4.5			SSP5-8.5	
ID	FID	Lat	Lon	Low	Middle	High	Low	Middle	High
1	172	42.05	72.82	26.06%	14.55%	32.73%	6.67%	100.00%	32.12%
2	173	41.87	73.56	20.00%	23.64%	31.52%	24.24%	23.03%	0.00%
3	168	41.45	73.67	51.52%	40.00%	63.03%	35.76%	37.58%	41.82%
4	179	41.38	73.65	0.00%	0.00%	20.00%	0.00%	0.00%	0.00%
5	186	41.40	73.69	0.00%	0.00%	0.00%	100.00%	0.00%	0.00%
6	166	41.77	74.71	69.70%	85.45%	90.30%	100.00%	52.12%	49.70%
7	183	41.60	74.89	0.00%	0.00%	0.00%	0.00%	0.00%	0.00%
8	160	41.63	74.98	100.00%	100.00%	100.00%	100.00%	100.00%	95.15%
9	162	41.15	75.23	100.00%	54.55%	100.00%	66.06%	81.82%	100.00%
10	167	41.64	75.56	100.00%	100.00%	95.15%	44.24%	98.18%	100.00%
11	170	41.63	75.56	54.55%	32.73%	69.09%	13.94%	24.24%	27.88%
12	127	39.62	71.95	100.00%	98.79%	90.30%	100.00%	89.70%	36.97%
13	68	39.63	71.91	100.00%	100.00%	97.58%	73.33%	70.91%	75.15%
14	11	39.60	71.88	100.00%	100.00%	98.18%	100.00%	84.24%	100.00%
15	155	39.43	72.13	11.52%	89.70%	0.00%	0.00%	0.00%	0.00%
16	146	39.32	72.25	15.76%	0.00%	23.03%	0.00%	0.00%	0.00%
17	112	39.37	71.80	100.00%	68.48%	100.00%	50.91%	75.76%	70.91%
18	138	39.32	71.73	100.00%	100.00%	100.00%	100.00%	100.00%	100.00%
19	48	39.70	71.69	100.00%	100.00%	100.00%	100.00%	94.55%	100.00%
20	72	39.70	71.66	99.39%	100.00%	100.00%	81.82%	86.67%	83.64%
21	82	39.69	71.63	100.00%	100.00%	100.00%	100.00%	98.18%	100.00%
22	40	39.48	71.43	100.00%	100.00%	100.00%	100.00%	100.00%	100.00%
23	144	39.32	71.33	100.00%	100.00%	100.00%	75.15%	100.00%	100.00%
24	156	39.36	71.20	100.00%	100.00%	100.00%	74.55%	92.12%	84.85%
25	107	39.03	71.27	100.00%	100.00%	100.00%	100.00%	100.00%	99.39%
26	31	39.05	71.18	100.00%	100.00%	100.00%	100.00%	100.00%	100.00%
27	8	39.03	71.10	100.00%	100.00%	100.00%	100.00%	100.00%	100.00%
28	85	39.26	70.73	100.00%	100.00%	99.39%	100.00%	100.00%	86.06%
29	118	39.35	70.62	100.00%	100.00%	100.00%	100.00%	100.00%	100.00%
30	109	39.24	70.06	100.00%	100.00%	100.00%	100.00%	100.00%	100.00%
31	73	39.22	70.10	100.00%	100.00%	100.00%	100.00%	100.00%	100.00%
32	94	38.63	71.31	99.39%	98.18%	100.00%	100.00%	100.00%	100.00%
33	95	38.63	71.47	83.64%	99.39%	100.00%	100.00%	100.00%	100.00%
34	65	38.65	71.32	100.00%	81.82%	100.00%	100.00%	100.00%	100.00%
35	69	38.68	71.44	100.00%	100.00%	99.39%	100.00%	100.00%	97.58%
36	60	38.68	71.43	100.00%	100.00%	100.00%	100.00%	100.00%	100.00%
37	129	38.69	70.71	92.12%	98.79%	99.39%	84.85%	100.00%	98.18%

FID is the ID of seasonal pumped hydropower storage potential sites in Hunt et al. [6], with IDs 1–11 located in the Syr Darya Basin and IDs 12–37 in the Amu Darya Basin.

REFERENCE:

1. Ma Y., Li Y., Wang H., et al. (2024). Non-deterministic multi-level model for planning water-ecology nexus system under climate change. *ll Rep. Sustain.* **1**:100032. DOI:10.1016/j.crsus.2024.100032
2. Liu S., Luo G. and Wang H. (2020). Temporal and Spatial Changes in Crop Water Use Efficiency in Central Asia from 1960 to 2016. *Sustainability* **12**:572. DOI:10.3390/su12020572
3. Wang X., Zhang J., Wang S., et al. (2023). Reviving the Aral Sea: A Hydro-Eco-Social Perspective. *Earth's Future* **11**:e2023EF003657. DOI:10.1029/2023ef003657
4. Geressu R. T., Siderius C., Rao Kolusu S., et al. (2022). Evaluating the sensitivity of robust water resource interventions to climate change scenarios. *Clim. Risk Manage.* **37**. DOI:10.1016/j.crm.2022.100442
5. Wang F., Huang G. H., Fan Y., et al. (2020). Robust Subsampling ANOVA Methods for Sensitivity Analysis of Water Resource and Environmental Models. *Water Resour. Manage.* **34**:3199-3217. DOI:10.1007/s11269-020-02608-2
6. Hunt J. D., Byers E., Wada Y., et al. (2020). Global resource potential of seasonal pumped hydropower storage for energy and water storage. *Nat. Commun.* **11**:947. DOI:10.1038/s41467-020-14555-y
