# Supplementary material for: A transboundary water allocation strategy for the Aral Sea Basin: Integrating the water-food-energy-environment nexus
Source: Innovation (Camb). 2026 Jan 7;7(5):101257. doi: 10.1016/j.xinn.2026.101257 (PMC13147992; doi:10.1016/j.xinn.2026.101257)
Supplement: Document S2. Figures S1 and S2 [file mmc2.pdf]

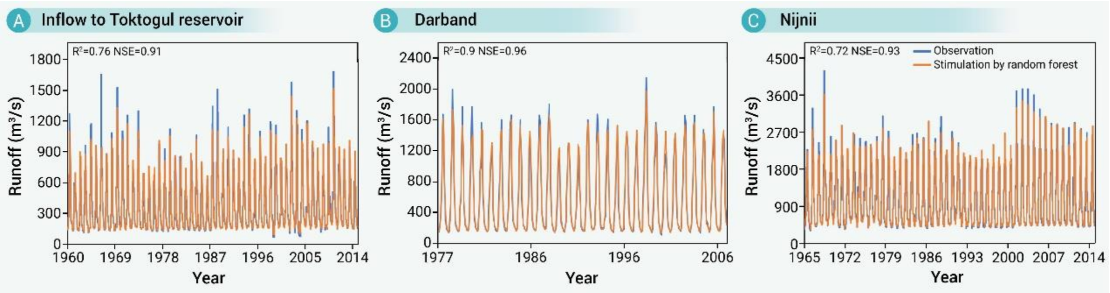

Fig. S1. Comparison and validation of observed and simulated runoff in the Aral Sea Basin

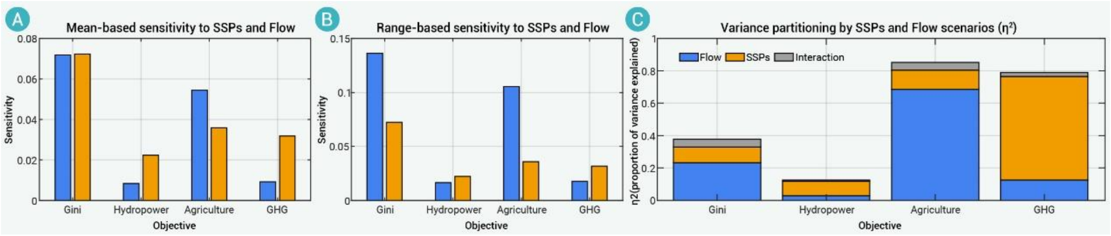

Fig. S2. Sensitivity analysis and ANOVA under SSPs and flow scenarios
