# Supplementary material for: A transboundary water allocation strategy for the Aral Sea Basin: Integrating the water-food-energy-environment nexus
Source: Innovation (Camb). 2026 Jan 7;7(5):101257. doi: 10.1016/j.xinn.2026.101257 (PMC13147992; doi:10.1016/j.xinn.2026.101257)
Supplement: Document S3. Article plus supplemental information [file mmc3.pdf]

ARTICLE Geographical Sciences

OPEN ACCESS

www.the-innovation.org

A transboundary water allocation strategy for the Aral Sea Basin: Integrating the water-food-energy-environment nexus

Yanan Hu,[1,2,7,9,10] Guangdong Sun,[3] Weili Duan,[1,2,4,5,*] Shan Zou,[1,2,6] Yanfeng Di,[1,2] Yaning Chen,[1] Patient Mindje Kayumba,[1,2] Wei Wei,[1,2] Philippe De Maeyer,[1,2,8,9,10] and Peter L.M. Goethals[7]

*Correspondence: duanweili@ms.xjb.ac.cn

Received: July 13, 2025; Accepted: December 31, 2025; Published Online: January 7, 2026; https://doi.org/10.1016/j.xinn.2026.101257

© 2026 The Author(s). Published by Elsevier Inc. on behalf of Youth Innovation Co., Ltd. This is an open access article under the CC BY license (http://creativecommons.org/licenses/by/4.0/).

GRAPHICAL ABSTRACT

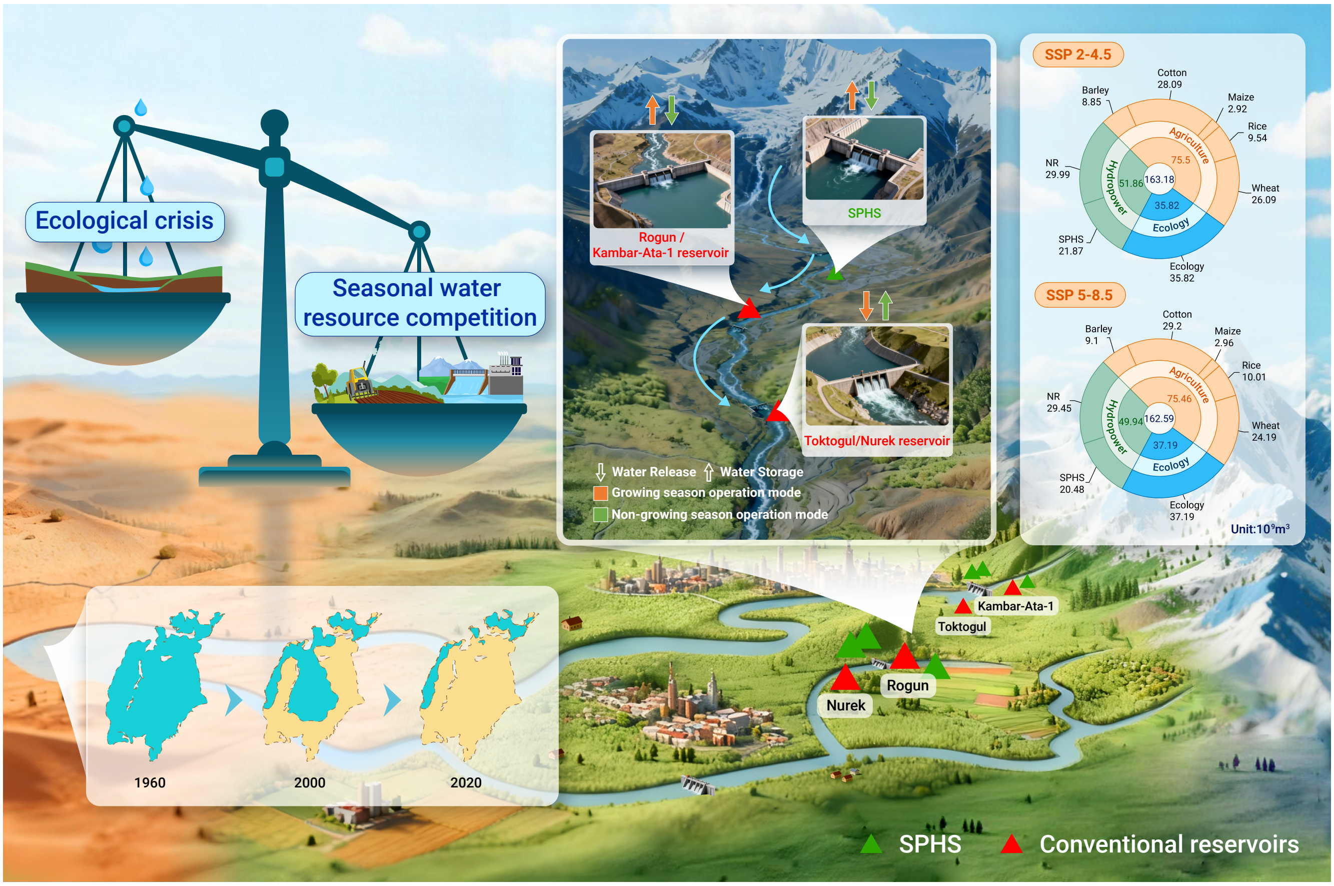

PUBLIC SUMMARY

- Coupling conventional reservoirs with seasonal pumped hydropower storage helps alleviate water competition.
- Water allocation optimization achieved relative equity (Gini < 0.29), balancing food, hydropower, and ecology.
- Trade-offs exist between benefits and water equity, while benefits are related to greenhouse gas emissions.

A transboundary water allocation strategy for the Aral Sea Basin: Integrating the water-food-energy-environment nexus

Yanan Hu,[1,2,7,9,10] Guangdong Sun,[3] Weili Duan,[1,2,4,5,*] Shan Zou,[1,2,6] Yanfeng Di,[1,2] Yaning Chen,[1] Patient Mindje Kayumba,[1,2] Wei Wei,[1,2] Philippe De Maeyer,[1,2,8,9,10] and Peter L.M. Goethals[7]

[1]State Key Laboratory of Ecological Safety and Sustainable Development in Arid Lands, Xinjiang Institute of Ecology and Geography, Chinese Academy of Sciences, Urumqi 830011, China
[2]University of Chinese Academy of Sciences, Beijing 100049, China
[3]Xinjiang Tarim River Basin Authority of China, Korla, Xinjiang 841000, China
[4]Xinjiang Key Laboratory of Water Cycle and Utilization in Arid Zone, Xinjiang Institute of Ecology and Geography, Chinese Academy of Sciences, Urumqi 830011, China
[5]Ili Station for Watershed Ecosystem Research, Chinese Academy of Sciences, Xinyuan 835800, China
[6]Aksu National Station of Observation and Research for Oasis Agro-ecosystem, Aksu 843017, China
[7]Department of Animal Sciences and Aquatic Ecology, Ghent University, 9000 Ghent, Belgium
[8]Department of Geography, Ghent University, 9000 Ghent, Belgium
[9]Sino-Belgian Joint Laboratory for Geo-Information, 9000 Ghent, Belgium
[10]Sino-Belgian Joint Laboratory for Geo-Information, Urumqi 830011, China
*Correspondence: duanweili@ms.xjb.ac.cn

Received: July 13, 2025; Accepted: December 31, 2025; Published Online: January 7, 2026; https://doi.org/10.1016/j.xinn.2026.101257
© 2026 The Author(s). Published by Elsevier Inc. on behalf of Youth Innovation Co., Ltd. This is an open access article under the CC BY license (http://creativecommons.org/licenses/by/4.0/).
Citation: Hu Y., Sun G., Duan W., et al., (2026). A transboundary water allocation strategy for the Aral Sea Basin: Integrating the water-food-energy-environment nexus. The Innovation **7(5)**, 101257.

Water resource competition has disrupted sustainable development in the Aral Sea Basin, necessitating integrated strategies for the water-food-energy-environment nexus to address challenges from ongoing climate change, ecological restoration, growing food demand, and potential hydropower projects impacting water stability. This study developed a multi-objective optimization model to address these issues. Results showed relatively equitable water allocation, with Gini coefficients consistently below 0.29 across all scenarios. Agricultural water use ranged from 71.71 to 80.53 $\times$ 10^9 m^3, while seasonal pumped hydropower storage reservoirs increased upstream controllable water to 42.91–58.47 $\times$ 10^9 m^3 (35%–44%). Hydropower remained stable owing to reservoir coordination. However, to ensure ecological flows (35.38–37.78 $\times$ 10^9 m^3), crop areas should be reduced by 14.37%–21.05% under SSP2-4.5 and 16.16%–23.93% under SSP5-8.5. A trade-off emerged between benefits and water allocation equity, particularly in high-emission, low-inflow scenarios, alongside a positive correlation between benefits and greenhouse gas emissions. These findings emphasize the critical need for integrated management of the Aral Sea Basin's interconnected resource systems.

INTRODUCTION

The 2030 global agenda for Sustainable Development Goals (SDGs) focused on the escalating crisis for resource security. Growing populations, urbanization, and climate change are increasing the demand for essential resources such as water, energy, and food, leading to their rapid depletion and compromising the critical interdependencies among them. Notably, global water demand, primarily for food and energy production, is projected to increase by 20%–30% by 2050, reaching 5,500–6,000 km^3 annually, far exceeding the current near-sustainable withdrawal of 4,600 km^3.[1] These interdependencies often create complex trade-offs, particularly at the river basins scale, where upstream regions often increase reservoir storage during summer for winter hydropower generation. This practice reduces the downstream irrigation supply causing seasonal water shortages and increased winter flood risk. Such imbalances in allocation exacerbate water scarcity, which already affects around 3.2 billion people, and contribute to estimated annual losses of $23.8 billion.[2]

These challenges are more pronounced in the transboundary Aral Sea Basin of Central Asia, spanning five countries. The upstream countries (Kyrgyzstan and Tajikistan) contribute about 70% of the water resources but have limited arable land and fossil fuels, leading to heavy reliance on hydropower. In contrast, the downstream countries (Kazakhstan, Turkmenistan, and Uzbekistan) hold 86% of the arable land yet access only 20% of the water resources, making their agriculture-based economies highly dependent on transboundary flows.[3] During the Soviet era, this conflict was managed through centralized water-energy trade agreements where upstream countries provided summer irrigation water in return for winter energy supplies from downstream countries. However, the collapse of the Soviet Union and the dismantling of the treaty intensified conflict over transboundary resource allocation, significantly challenging regional water and energy security. The density of the water conflict network in Central Asia increased by 0.18, with the Aral Sea Basin experiencing the most intense disputes.[4] In this context, reservoir operations remain volatile—Toktogul's releases fluctuated by 31%–50% (2007–2012) and Nurek's inflow and release data showed similar variability, underscoring the absence of a rigid transboundary operational framework.[5] This is exemplified by Tajikistan's Rogun Dam project, which aims to achieve energy self-sufficiency through hydropower but may cut down downstream agricultural revenue by 37%.[6] Additionally, Afghanistan's significant hydropower potential of 23,000 MW, which includes the largest Amu Darya tributary (Pyanj River), could further alter regional water availability.[7] The agricultural water demand remains substantial, although cultivated land expansion slowed to 11.02–18.69 kha after the Soviet Union collapse.[8] Large-scale irrigation withdrawals drastically reduced inflows into the Aral Sea, leading to an approximate 87.85% loss in its volume (1,000.51 km^3).[9] This triggered a cascade of severe ecological problems that were intensified by climate change, thereby attracting global attention since the 1960s.[10] Notably, the region is warming at 0.36°C–0.42°C/decade, more than twice the global average, which is altering hydrological patterns.[3,11] While annual river runoff in the region may increase by 10% this century,[12] rising temperatures in the Amu Darya Basin are projected to boost crop water demand by 10.6%–16% by 2050.[13] Furthermore, seasonal flow patterns in the Syr Darya Basin may shift from spring/early summer to late winter/early spring, adversely affecting water availability during peak agricultural periods.[14,15] Consequently, the intensifying water competition between downstream irrigation and upstream hydropower generation, and between agricultural use and ecological needs, complicates transboundary water management in the climate change context. These scenarios highlight the urgency of addressing water-related challenges in the Aral Sea Basin. A comprehensive approach that integrates water, food, energy, and environmental management under diverse hydrological and socioeconomic scenarios is critical to mitigating resource competition and fostering sustainable development.

Numerous studies have tried to address the complex water and ecological challenges in the Aral Sea Basin through advanced modeling approaches, yet critical methodological and contextual gaps remain. For instance, Ma et al.[16] demonstrated that coordinated management via a bi-level decentralized chance-constrained programming model could increase ecological water allocation by 12.5%, albeit at the cost of reducing overall system benefits. Zhang et al.,[17] using a copula-based stochastic fractional programming model, projected that agricultural water use would decline from 69.1% in 2021 to 53.9% by 2035, necessitating a 16.4% expansion in wheat cultivation to maintain food security. By incorporating climate change scenarios, Ruan et al.[18] found that optimizing wheat cultivation could save 28.7 mm of water under warming of 0.51°C–0.91°C, while Ma et al.[19] proposed a multi-level chance-constrained fuzzy programming model to jointly sustain the water-ecology nexus in Central Asia, and emphasized increasing ecological water quotas to achieve systemic

balance. However, most existing studies rely on deterministic or single-solution planning approaches, which yield only point solutions based on predefined preferences and fail to characterize the full trade-off frontier among competing objectives such as energy production, irrigation needs, and ecosystem requirements. This gap is substantiated by the frequent overlooking of transboundary allocation equity between upstream and downstream countries, and the exclusion of impacts from emerging infrastructure, particularly the Rogun Dam (13.3 km^3) and Kambar-Ata-1 Dam (5.4 km^3). These projects are expected to significantly alter regional hydrological regimes and sectoral water allocation. For example, the Kambar-Ata-1 project, agreed upon by the energy and water authorities of Kazakhstan, Kyrgyzstan, and Uzbekistan, may shift Toktogul Dam's operation from primarily power generation to irrigation. Furthermore, although the mountainous regions show considerable potential for seasonal pumped hydropower storage (SPHS),[20] existing research does not systematically explore its optimal coordination with conventional reservoirs, especially under climate-induced water variability, a critical gap exists, as Zakeri et al.[21] found the system to be unsustainable with just a 20% change in water supply or demand, indicating the need for more resilient and adaptive water-energy strategies amid growing climate and demand pressures.

Therefore, we aim to address these gaps by developing a multi-objective optimization framework that integrates agricultural, hydropower energy, and ecological water demands while synergistically considering transboundary equity, climate scenarios, and the operational potential of SPHS and the projected conventional reservoirs. Specifically, this study seeks to: (1) develop a water-energy optimization scheme to abate the seasonal water competition through effective coordination of SPHS and conventional reservoirs operation and (2) apply the multi-objective algorithm for improved water allocation equity, agricultural productivity, hydropower generation, and greenhouse gas (GHG) emission under multiple scenarios. This would facilitate a systematic characterization of cross-sectoral trade-offs and informed strategies regarding the selection of policy-relevant water allocation for sustainable basin management.

MATERIALS AND METHODS

Data

Historical inflow data for the Naryn, Vakhsh, and Pyanj Rivers were obtained from runoff monitoring stations (inflow to Toktogul Reservoir, Darband, and Nijnii stations).[22-24] Future runoff projections were derived using climate data from NASA's Earth Exchange Global Daily Downscaled Projections (NEX-GDDP-CMIP6). This dataset applies bias-corrected spatial disaggregation to CMIP6 data, offers higher resolution, and incorporates local topographical factors affecting hydrometeorological events, with extensive applications at regional, national, and basin scales.[25] The study focused on two shared socioeconomic pathways (SSPs) scenarios: SSP2-4.5 (moderate mitigation) and SSP5-8.5 (high forcing without mitigation). Specifically, SSP2-4.5 reflects a moderate emission pathway, assuming that some mitigation measures are taken, and is aligned with the goal of limiting global warming to around 2°C by the end of the century. Conversely, SSP5-8.5 represents a high-emission scenario with minimal mitigation, leading to a potential warming of nearly 5°C. These two scenarios present a clear range of possible futures—from a relatively optimistic case to a worst-case scenario. We used historical data (1960–2014) and future projections (2015–2050) from 11 models with 8 hydrometeorological variables based on the first ensemble member (r1i1p1f1) (Table S1). The results are reported as the median of the multi-model ensemble, thereby providing a robust representation of hydrometeorological characteristics while mitigating biases from individual models.

Potential evapotranspiration data for future crop water demand were sourced from Bjarke et al.[26] using the median of multi-model ensembles. Owing to the absence of future crop area data for the Aral Sea Basin, 2015 data from GAEZ+_2015[27] were used for harvested area and yields of cotton, maize, wheat, barley, and rice. These five crops together account for approximately 80% of the total harvested area in Central Asia. Future water demands in the Aral Sea Basin for industrial, municipal, and livestock purposes under different scenarios were derived from Khan et al.[28] Ecological water demand data for 2050 were sourced from Wang et al.,[12] while population data were obtained from the Socioeconomic Data and Applications Center (https://sedac.ciesin.columbia.edu/). The per capita food calorie requirements under different SSPs were obtained from van Dijk et al.[29] Data on hydropower in Central Asia in 2050 were derived from the 2022 Central Asia Water Yearbook (http://www.cawater-info.net/yearbook/). The hydropower demand of the Aral Sea Basin was estimated using electricity consumption data from 2015,[30] per capita electricity consumption data, and 2050 population projections under different SSPs.

Crop prices and agricultural input costs were assumed to remain at current levels and were sourced from the Food and Agriculture Organization (FAO), the International Fertilizer Association, Wichelns et al.,[31] the Times of Central Asia (https://timesca.com/turkmenistans-government-supplied-agricultural-services-soar-in-cost/), and Kazakhstan's Ministry of Water Resources and Irrigation (https://agrosektor.kz/agriculture-news/turkestanskim-agrariyam-snizili-ceny-na-polivnuyu-vodu-do-38.html). The residential and commercial electricity prices in Central Asia were sourced from He et al.,[32] the Asian Development Bank,[33] and Global Petrol Prices (https://www.globalpetrolprices.com/electricity_prices/). The crop calorie content data were obtained from Ray et al.[34] The SPHS data were sourced from Hunt et al.,[20] and reservoir hydropower efficiency data were drawn from Akhmedov and Petrov[35] and Normatov.[36]

Method

This study assesses future water supply and demand (2015–2050) in the Aral Sea Basin using Random Forest and CROPWAT models. Random Forest is a non-parametric ensemble model that captures nonlinear relationships between hydrometeorological variables and runoff and performs well with limited data, making it suitable for data-scarce regions such as Central Asia.[19,37,38] Its bootstrap aggregation mechanism enhances model stability and generalizability. CROPWAT, developed by FAO and based on the Penman-Monteith equation, is widely used for estimating crop water and irrigation demands. With relatively low data requirements and global applicability, it can flexibly simulate irrigation demand under varying climate, soil, and crop management scenarios, providing a consistent and transparent framework for large-scale assessments. The combination of Random Forest and CROPWAT feasibly links the supply and demand spectrum of the water system in an integrated framework, enabling comprehensive assessment of future water allocation strategies under climate change. The calculation protocols and formulations for both Random Forest and CROPWAT are detailed in supplemental information Text S1 and Text S2. In addition, to address uncertainties arising from climate change and economic development, this study designed six scenario settings. Specifically, three inflow conditions were defined using the Pearson type III distribution to represent high (25%), medium (50%), and low (75%) water availability. These were further combined with two SSPs selected for their representativeness and contrast. Together, these combinations resulted in six scenarios (SSP2-4.5 High, SSP2-4.5 Medium, SSP2-4.5 Low, SSP5-8.5 High, SSP5-8.5 Medium, and SSP5-8.5 Low), which were subsequently used as input data for the water resource optimization model.

To encompass the basin's complex water management challenges and ensure ecological sustainability, a multi-objective optimization model was employed to address the water allocation issue. The model incorporated four objective functions: (1) maximizing water allocation equity, (2) maximizing hydropower benefits, (3) maximizing agricultural productivity benefits, and (4) minimizing GHG emissions from agriculture. Multiple constraints were applied to ensure practicality and feasibility, including total water balance, seasonal water balance, land use limitations, food security, hydropower requirements, ecological water demand of the Aral Sea, reservoir capacity, and non-negativity constraints. These constraints enable the model to effectively balance competing demands and address the basin's multifaceted challenges. The nomenclature for parameters and variables is described in Table S2.

(1) Maximizing water allocation equity

The sustainable allocation of water resources in the Aral Sea Basin requires equitable and reasonable allocation among riparian countries. This study defined the equity objective based on per capita controllable water and measures cross-country disparities using the Gini coefficient. The Gini coefficient, with its anonymity and scale-invariance properties, captures overall imbalance without imposing subjective weights. Adopting a per capita metric regulates heterogeneity in population size, thereby enhancing the interpretability of "equity" in demographic terms. Subject to constraints on total and seasonal water availability, ecological protection, food security, and hydropower electricity supply, minimizing the Gini coefficient steers per capita allocations toward balance and advances the goal of an equitable and reasonable allocation. Therefore, minimizing the Gini coefficient can be expressed as:

$$\text{Min } Gini = \frac{1}{2I\sum_{i=1}^{I}\frac{W_i}{PO_i}} * \sum_{p=1}^{I}\sum_{q=1}^{I}\left|\frac{W_p}{PO_p} - \frac{W_q}{PO_q}\right| \quad \text{(Equation 1)}$$

(2) Maximizing hydropower benefits

Hydropower, as a key renewable energy source, contributes to energy security (SDG7) and global warming mitigation while providing energy source for the two upstream

countries in the Aral Sea Basin, thereby facilitating economic development. The objective function was grounded in water-to-electricity conversion and tiered electricity pricing: hydropower from conventional reservoirs is credited by first meeting residential load at the residential tariff, with any remaining energy sold at the commercial tariff; hydropower-generated by SPHS was settled at the commercial tariff, and SPHS costs provided by the database were included on the cost side.[20] This formulation ensured dimensionally consistent revenue accounting and the temporal allocation of water, thereby maximizing the net benefits of the hydropower sector. Therefore, maximizing hydropower benefits can be expressed as:

$$\text{Max } \mathit{Hydropower\ Benefits} = \sum_{i=1}^{I}\left(\sum_{j=1}^{J} P_{hi} * HEC_i + P_{bi} * \left(HW_j * WA_{jh} - HEC_i\right) + \sum_{k=1}^{K}\left(P_{bi} * E_k - C_k\right)\right) \quad \text{(Equation 2)}$$

(3) Maximizing agricultural productivity benefits

Rapid population growth, industrialization, and urbanization have significantly increased the food demand, which needs to be met through sufficient agricultural production. Agriculture represents a crucial economic activity in the three downstream countries of the Aral Sea Basin. Agricultural economic benefits were evaluated under a consistent price-yield-cost monetary framework: revenue was computed as the product of crop price and output, and the input costs of irrigation water and nitrogen fertilizer were deducted. This objective guided economically rational trade-offs in crop structure and input use, prioritizing combinations with higher returns and superior water- and nitrogen-use efficiency, thereby achieving the agricultural sector's economic goals without violating intersectoral or ecological constraints. Therefore, maximizing the agricultural productivity benefits can be expressed as:

$$\text{Max } \mathit{Agricultural\ Benefits} = \sum_{i=1}^{I}\sum_{t=1}^{T}\left(P_{it} * A_{it} * Y_{it} - P_{it}^{irri} * A_{it} - P_{it}^{fert} * A_{it}\right) \quad \text{(Equation 3)}$$

(4) Minimizing agricultural GHG emissions

Agricultural activities generate substantial GHG emissions, exacerbating climate change. Reducing these emissions not only reverses global warming but also ensures agricultural sustainability, which is critical for environmental protection and long-term development in the Aral Sea Basin. In this study, GHG accounting followed the Intergovernmental Panel on Climate Change inventory approach: direct and indirect *N* emission factors were applied to nitrogen inputs, and methane from paddy rice was quantified using area-based emission factors. This formulation aligns with international practice, ensuring comparability and verifiability. When co-optimized with agricultural revenues, it encourages lower nitrogen application rate and a reduced share or improved spatiotemporal allocation of high-methane crops. This was done without materially compromising output, thereby reflecting the sector's mitigation orientation. Therefore, minimizing agricultural GHG emissions can be expressed as:

$$\text{Min } GHG = \sum_i^I \sum_t^T \left(\left(\left(A_{it} * N_i * EF_d + A_{it} * N_i * \left(F_{vol} * EF_{vol} + F_{leach} * EF_{leach}\right)\right) * \frac{44}{28} * 265\right) + A_i^{rice} * EF_{CH_4} * 28\right) \quad \text{(Equation 4)}$$

The constraint functions are shown as follows.

(1) Total water balance

Based on mass conservation, the total balance sums sectoral volumetric demands and caps them by total available water, keeping all optimal solutions within the physical supply limit.

$$\sum_i^I \left(\sum_t^T A_{it} * WD_t + WD_{io}\right) + WD_e + \leq TAW \quad \text{(Equation 5)}$$

(2) Seasonal water balance

The inflow (and stored water) during the growing and non-growing seasons must meet the water demands for crop growth, as well as for industrial, domestic, and livestock needs. It avoids strategies that are feasible only on an annual basis but inapplicable within seasons. It also ensures that sectoral demands in each season do not exceed seasonal availability.

$$\sum_x^X \sum_i^I \sum_t^T A_{it} * WD_{xt} + WD_{xio} \leq SAW \quad \text{(Equation 6)}$$

(3) Land area

The arable land constraint reflects land endowments and the recent stability of cultivated areas. Given that the cultivated area in the Aral Sea Basin has remained stable recently,[8,39] this study held total cropland at its current level and optimized the crop structure within this limit, thereby confining expansion pressure to feasible limits and directing gains primarily toward structural and efficiency improvements rather than unconstrained scale growth.

$$\sum_i^I \sum_t^T A_{it} \leq A \quad \text{(Equation 7)}$$

(4) Food security

The production of food crops must meet the food demands of the regional population. This criterion ensures that the goal of agricultural economic returns does not compromise adequate staple food supply, thereby maintaining a basic level of social welfare at the institutional level.

$$\sum_i^I \sum_t^T A_{it} * Y_{it} \geq \sum_i^I FD * PO_i \quad \text{(Equation 8)}$$

(5) Hydropower demand

The electricity supply constraint reflects the planning adequacy or scenario-based demand floor. Together with the seasonal balance and reservoir dynamics, it ensures that allocations to agriculture and ecology do not erode the power system's basic supply capability.

$$\sum_{j=1}^{J}\left(HW_j * WA_{jh}\right) + \sum_{k=1}^{K} E_k \geq HD \quad \text{(Equation 9)}$$

(6) Ecological water demand of the Aral Sea

The ecological water constraint is anchored to the Aral Sea's target level (e.g., +40 m), thereby internalizing the ecological safety threshold as an optimization boundary. Ecological water supply should be adequate enough to meet the ecological demand, ensuring that productivity does not overlook ecosystem needs. Empirical evidence indicates that restoring the Aral Sea to +40 m is feasible under various water-use and management reform scenarios.[12] This restoration would restrict the salinity to a tolerable range and reduce the risk of sandstorms by 58%. Accordingly, all scenarios in this study adopted the +40 m benchmark to set ecological water supply requirements.

$$WS_e \geq WD_e \quad \text{(Equation 10)}$$

(7) Reservoir capacity

The storage volume of reservoirs must not exceed their designed maximum capacity or fall below their dead storage capacity. Together with the seasonal water balance, this dynamic system determines storage-regulation capacity, thereby affecting the temporal feasibility of hydropower generation, ecological releases, and agricultural irrigation.

$$V_{min} \leq V \leq V_{max} \quad \text{(Equation 11)}$$

(8) Non-negativity constraint

All decision variables in the model must be non-negative.

$$A_{it} \geq 0 \quad \text{(Equation 12)}$$

$$k \geq 0 \quad \text{(Equation 13)}$$

The NSGA-III algorithm introduced a unique selection mechanism and reference point strategy, which significantly enhances the convergence of Pareto-optimal solutions. To further improve the distribution of solutions, the algorithm incorporates association and niching operations that prevent clustering in specific regions of the Pareto front, thereby maintaining overall diversity.[40] These mechanisms make the

NSGA-III algorithm particularly effective for resolving multi-objective optimization problems (≥3 objectives). In the context of this research, which involves four conflicting objectives and a large number of decision variables, such properties are invaluable. By ensuring both convergence and a well-distributed set of solutions across the Pareto front, the NSGA-III algorithm can provide decision-makers with a balanced portfolio of trade-off alternatives. Moreover, compared with other representative algorithms such as RVEA and MOEA/D, the NSGA-III algorithm is relatively parameter-light and robust, facilitating reproducibility and ensuring more stable computational efficiency under multiple scenarios.[41] This combination of robust convergence, diversity preservation, and computational efficiency makes the NSGA-III algorithm particularly suitable for the water-food-energy-environment nexus optimization problem. Therefore, the NSGA-III algorithm was implemented through the PlatEMO platform to solve the proposed multi-objective optimization model. The solution process was as follows.

(1) Parameter initialization

The population size was set to 200, crossover probability to 0.9, mutation rate to 0.01, and maximum generations to 50,000. These parameters balanced search pressure, diversity, uniformity, and convergence speed. In the PlatEMO implementation of NSGA-III, the effective population size was internally aligned with the number of reference points. In addition, a hybrid encoding scheme (real and binary) enhances adaptability to practical problems.

(2) Reference point generation

Uniform reference points were generated automatically by the algorithm implementation. Given the number of objectives M and the number of equal divisions per objective axis H, the number of reference points is calculated by the following:

$$N_r = \binom{M + H - 1}{H} \quad \text{(Equation 14)}$$

where N_r is the number of reference points. This ensures solution diversified for NSGA-III.

(3) Initial population generation

Initial population N was randomly generated, which consisted of individuals defined by decision variables within feasible ranges.

(4) Non-dominated sorting

Individuals were classified into non-dominated fronts based on dominance relationships.

(5) Association with reference points

Euclidean distances were computed between individuals and reference points. Each individual was associated with its nearest reference point and the number of individuals linked to each point was recorded.

(6) Selection

Individuals linked to underrepresented reference points were prioritized. For points with multiple associated individuals, the one closest to the point was selected. If additional individuals were needed, this process was repeated with the next non-dominated front.

(7) Crossover and mutation

Crossover and mutation operators were applied to generate offspring solutions.

(8) Next-generation formation

Offspring were combined with the current population using an elitist strategy to retain high-quality individuals for the next generation.

(9) Termination

When the termination criteria (e.g., maximum iterations or convergence) were met, the algorithm was terminated. Otherwise, step 5 was repeated.

RESULTS

Development of the water-energy coordination scheme in the Aral Sea Basin

To address the complexity of seasonal water demand in the Aral Sea Basin, we developed an integrated water-energy coordination scheme (Figure 1). The scheme was based on two principal systems: the Naryn River within the Syr Darya Basin, utilizing Toktogul and Kambar-Ata-1 reservoirs, and the Vakhsh River within the Amu Darya Basin, utilizing the Nurek and Rogun reservoirs. In each system, the lower reservoir (Toktogul and Nurek) functioned as a counter regulation system for its upper counterpart (Kambar-Ata-1 and Rogun), with both systems complemented by numerous SPHS reservoirs. During the growing season, the primary water demand was for agriculture. The lower Toktogul and Nurek reservoirs released their stored water to meet irrigation demand while concurrently generating power. Since energy demand was lower in this season, surplus electricity was used to pump excess water from the Vakhsh River and Naryn River, directing them to their respective SPHS reservoirs. This was facilitated by the prior filling of the Toktogul and Nurek reservoirs during the non-growing season, which ensured that irrigation during the growing season did not depend on new inflows. Simultaneously, new inflows from glacial melt and precipitation were captured and stored in the Kambar-Ata-1 and Rogun reservoirs, as well as SPHS reservoirs. During the non-growing season, the operational focus shifted to meeting electricity demand. The stored water in the upper reservoirs was released for hydropower generation. This process ensured that these counter regulation reservoirs were refilled and equipped with the required water volume to support irrigation in the subsequent growing season, thereby mitigating seasonal water demand imbalances.

Furthermore, the study addressed potential water scarcity from Afghanistan's Kosh tepa project, which may reduce summer inflows to the Panj River (Amu Darya Basin) by 15%.[42] We modeled this reduction on the Panj River, a key tributary providing approximately 80% of the Amu Darya's flow alongside the Vakhsh River.[6] The Naryn River contributed to approximately 40% of the Syr Darya's flow.[43] The other rivers within the Aral Sea Basin were assumed to maintain normal hydrological patterns. Our analysis optimized the basin's water resources through restructuring agricultural crop patterns for efficiency and synergizing conventional reservoirs with SPHS reservoirs. This approach aimed to balance competing water demands between irrigation, energy production, and ecological need, thereby ensuring a fair, efficient, and sustainable allocation for the region.

Equity in water resource allocation in the Aral Sea Basin

Using the Gini coefficient as an equity indicator, we evaluated the proposed integration of conventional and SPHS reservoirs in the Aral Sea Basin, and assessed its role in balancing water allocation, ensuring coordination, and promoting sustainable development. Figure 2 shows that the Gini coefficients remained consistently below 0.29 across all SSPs and inflow scenarios. Considering that a Gini coefficient of 0.4 is internationally recognized as the "warning line" for resource allocation gaps,[4] these results indicate a relatively high level of water allocation equity within the basin while ensuring economic development and ecological wellbeing.

The analysis revealed a direct correlation between water availability and equity. Specifically, high-inflow scenarios exhibited lower Gini coefficients, reflecting a more equitable allocation in wet years. Conversely, low-inflow scenarios showed higher coefficients, indicating reduced equity under water scarcity. This pattern, where scarcity intensifies allocation challenges, was demonstrated in other arid systems. For instance, in China's Shule River Basin, a 75% water supply assurance rate reduced overall supply capacity by 50%, causing agricultural supply to fall below minimum requirements.[44] Within the Aral Sea Basin, the high-emission SSP5-8.5 scenario introduced greater variability in equity. Gini coefficients ranged more widely (0.15–0.29) compared with the more stable range under the SSP2-4.5 scenario (0.16–0.25). This suggests increased uncertainty for equitable water allocation under high-emission scenarios, a problem linked to its impact on hydrology. Rising temperatures trigger earlier glacier and snowmelt episodes and reduce summer precipitation, thereby altering runoff regimes and causing increased spring runoff and peak flows to occur up to 1 month earlier.[45] Such shifts generated an anomaly between water supply timing and demand, further complicating the coordination among agricultural irrigation, hydropower generation, and ecological water

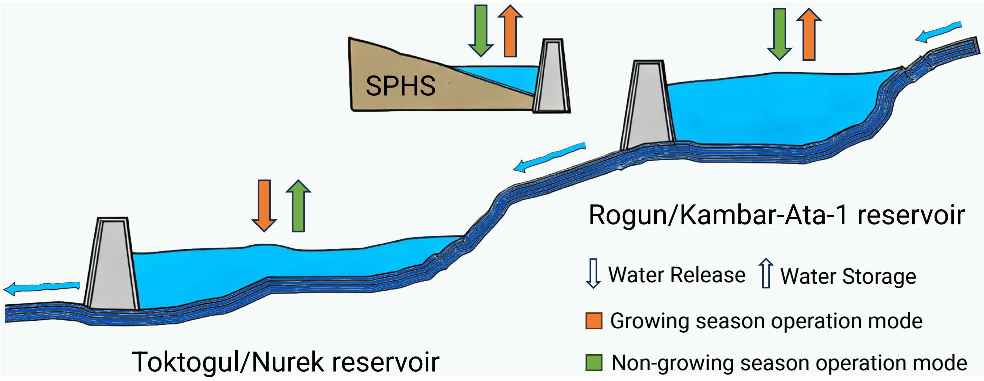

Figure 1. Seasonal water flow patterns for conventional and SPHS reservoirs in the Aral Sea Basin

requirements. In contrast, the more moderate SSP2-4.5 scenario appears to alleviate these pressures, promoting more predictable and stable water allocation outcomes.

Optimized water allocation strategies in the Aral Sea Basin

Agricultural crop structure adjustment and water allocation strategies. Proper adjustments to agricultural crop structures can improve regional water allocation equity and foster economic development. Table 1 presents the optimized crop planting structures, with values showing the minimum-maximum range within each SSPs and inflow scenario. Overall, crop planting areas ranged from 5,160.32 to 5,595.79 × 10^3 ha under the SSP2-4.5 scenario and 4,972.09 to 5,480.07 × 10^3 ha under the SSP5-8.5 scenario. Compared with the SSP5-8.5 scenario, SSP2-4.5 was associated with a relatively higher population in Central Asia, which in turn drives a greater demand for food, water, and other essential resources. In contrast, the SSP5-8.5 scenario projected a smaller population, as declining fertility rates are driven by higher living standards, greater educational attainment (particularly among women), and severe environmental stress, including intensified water scarcity and land degradation, despite rapid economic growth and urbanization.[46,47] Moreover, as rural livelihoods became increasingly unsustainable owing to environmental degradation, there was a marked trend of out-migration from environmentally stressed rural areas toward urban centers or even across national borders.[25] In addition, under the SSP5-8.5 scenario, the ecological water demand for the Aral Sea was greater than that under SSP2-4.5,[12] further limiting the water resources available for agricultural irrigation.

Within the crop structure, wheat and cotton were dominant, followed by barley, rice, and maize. Notably, wheat, critical for food security, covered 2,351.06–2,475.94 × 10^3 ha under the SSP2-4.5 scenario, compared with 2,088.04–2,263.21 × 10^3 ha under SSP5-8.5. Barley and maize exhibited less variability in their projected planting areas. Under SSP2-4.5 the areas were 720.95–883.28 × 10^3 and 201.59–261.11 × 10^3 ha, respectively. While, under the SSP5-8.5 scenario, the ranges were 734.17–854.76 × 10^3 and 194.82–268.45 × 10^3 ha, respectively. Cotton, a high-value crop, occupied more area under the SSP5-8.5 scenario (1,491.51–1,691.12 × 10^3 ha) than in the SSP2-4.5 scenario (1,461.02–1,613.01 × 10^3 ha). Similarly, rice, a high-water-demand crop primarily cultivated in well-irrigated areas, covered an area of 406.22–496.25 × 10^3 ha under the SSP5-8.5 scenario. This was slightly higher than 390.56–467.29 × 10^3 ha under SSP2-4.5. These trends followed cotton's profitability and the high price of rice was driven by its scarcity. Consequently, crop planting adjustments appeared to prioritize profitability while still ensuring food security.

Crop planting areas varied across different inflow scenarios, with larger areas observed during wet periods and smaller areas during dry periods. This indicated that the available runoff during irrigation significantly influenced planting. Notably, wheat and barley exhibited complementary fluctuations under medium inflow in SSP2-4.5, likely owing to their similar growth cycles and characteristics, such as strong drought tolerance. This resilience enables plants to maintain stability under limited water availability, minimizing yield variability. Such complementarity enhances the resilience of the agricultural system, enabling it to better cope with challenges such as climate change and water variability.[48]

Agricultural water allocation depends on planting area and irrigation water demand. Figure 3A shows agricultural water allocation under different SSPs and inflow scenarios in the Aral Sea Basin, ranging from 71.71 × 10^9 to 80.53 × 10^9 m^3. Similarly, Wang et al.[12] estimated that combining flood irrigation with drip irrigation could reduce water demand in the Aral Sea Basin to 78.1 × 10^9 m^3 between 2021 and 2050. Under the SSP2-4.5 scenario, agricultural water allocations were 72.31–72.59 × 10^9, 74.26–76.47 × 10^9, and 76.78–79.52 × 10^9 m^3 for low, medium, and high inflow, respectively. Under the SSP5-8.5 scenario, the corresponding allocations were 71.71–73.34 × 10^9, 74.10–75.99 × 10^9, and 76.49–80.53 × 10^9 m^3. Despite the smaller planting areas under the SSP5-8.5 scenario, water allocation was comparable with that under SSP2-4.5, which was attributed to increased areas of high-value but water-intensive crops, such as cotton and rice.

Specifically, for cotton (Figure 3C), allocation under the SSP5-8.5 scenarios increased to the ranges of 27.90–28.21 × 10^9, 29.30–29.44 × 10^9, and 28.50–31.41 × 10^9 m^3 for the low, medium, and high inflow, respectively, surpassing the respective SSP2-4.5 allocations of 27.02–27.06 × 10^9, 27.25–29.02 × 10^9, and 28.08–29.50 × 10^9 m^3. A parallel increment was observed for rice, with SSP5-8.5 showing allocation of 9.23–9.42 × 10^9, 9.52–9.54 × 10^9, and 10.65–11.36 × 10^9 m^3, which exceeded the corresponding SSP2-4.5 values of 8.76–8.83 × 10^9, 9.24–9.36 × 10^9, and 10.50–10.54 × 10^9 m^3 (Figure 3E). Conversely, wheat, the region's primary food crop experienced a marked reduction in water allocation under the SSP5-8.5 scenario, receiving 23.15–24.66 × 10^9, 23.85–25.27 × 10^9, and 23.96–25.53 × 10^9 m^3 for the three inflow conditions (Figure 3F). Under SSP2-4.5, it ranged from 26.10 to 26.26 × 10^9, 25.32 to 26.05 × 10^9, and 26.09 to 27.52 × 10^9 m^3, respectively. Additionally, the water for maize and barley showed slight fluctuation, ranging from 2.50 to 3.21 × 10^9 and 7.93 to

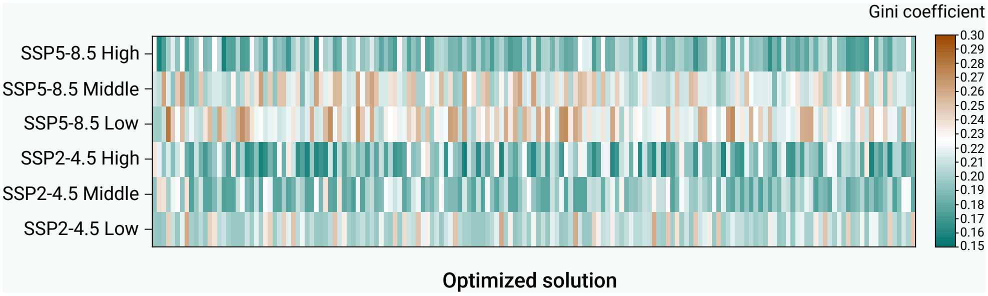

Figure 2. Gini coefficient of water resource allocation in the Aral Sea Basin under different SSPs and inflow scenarios The *x* axis represents different optimized solution, each corresponding to a Pareto-optimal water allocation strategy derived from the multi-objective optimization.

Table 1. Adjusted planting structures under different SSPs and inflow scenarios (unit: 1,000 ha)

		Total	Barley	Cotton	Maize	Rice	Wheat
SSP2-4.5	low min	5,160.32	720.95	1,461.02	201.59	390.56	2,385.34
	low max	5,193.72	725.30	1,464.12	204.56	394.22	2,411.31
	middle min	5,345.06	835.47	1,475.68	245.62	414.93	2,351.06
	middle max	5,502.66	883.28	1,584.81	257.59	420.43	2,415.30
	high min	5,359.77	786.33	1,497.88	256.74	465.44	2,348.37
	high max	5,595.79	821.15	1,613.01	261.11	467.29	2,475.94
SSP5-8.5	low min	4,972.09	734.17	1,491.51	194.82	406.22	2,089.34
	low max	5,117.36	793.51	1,518.00	224.64	415.69	2,213.25
	middle min	5,023.81	757.24	1,543.34	206.99	415.57	2,088.04
	middle max	5,177.61	787.74	1,554.66	234.33	416.38	2,193.87
	high min	5,177.13	766.64	1,499.67	249.76	463.40	2,102.57
	high max	5,480.07	854.76	1,691.12	268.45	496.25	2,263.21

9.30 × 10^9 m^3 under SSP2-4.5 and 2.48 to 3.40 × 10^9 and 8.41 to 10.00 × 10^9 m^3 under SSP5-8.5 (Figures 3D and 3B).

Coordination and water allocation strategies for conventional reservoirs and SPHS reservoirs

Seasonal variations in water cycle patterns of conventional reservoirs coupled with SPHS reservoirs can enhance upstream hydropower water allocation. In the Aral Sea Basin, the distribution of SPHS sites showed significant spatial variability, with higher selection probabilities in the Vakhsh River than in the Naryn River (Figures 4A–4F). Most SPHS reservoirs in the Vakhsh River Basin exhibited selection probabilities exceeding 68%, except for some that had lower probabilities due to their limited water storage and energy capacities (i.e., IDs 15 and 16; Table S3). Notably, the SPHS reservoirs (IDs 18, 22, 26, 27, 29, 30, and 31) were selected with 100% probability across all scenarios, characterized by high water storage and energy capacities combined with lower costs. In contrast, reservoirs (only IDs 6, 8, 9, and 10) in the Naryn River Basin exhibited selection probabilities exceeding 50% across scenarios, primarily attributed to their lower costs despite comparable energy capacities (Table S3). The runoff of the Vakhsh River exceeded that of the Naryn River, which partially influences the distribution of SPHS reservoirs. The selection of individual SPHS reservoirs was governed by their water storage and energy capacities as well as construction and operational costs. These factors should be comprehensively considered, prioritizing the sites with high selection probabilities as identified in this study.

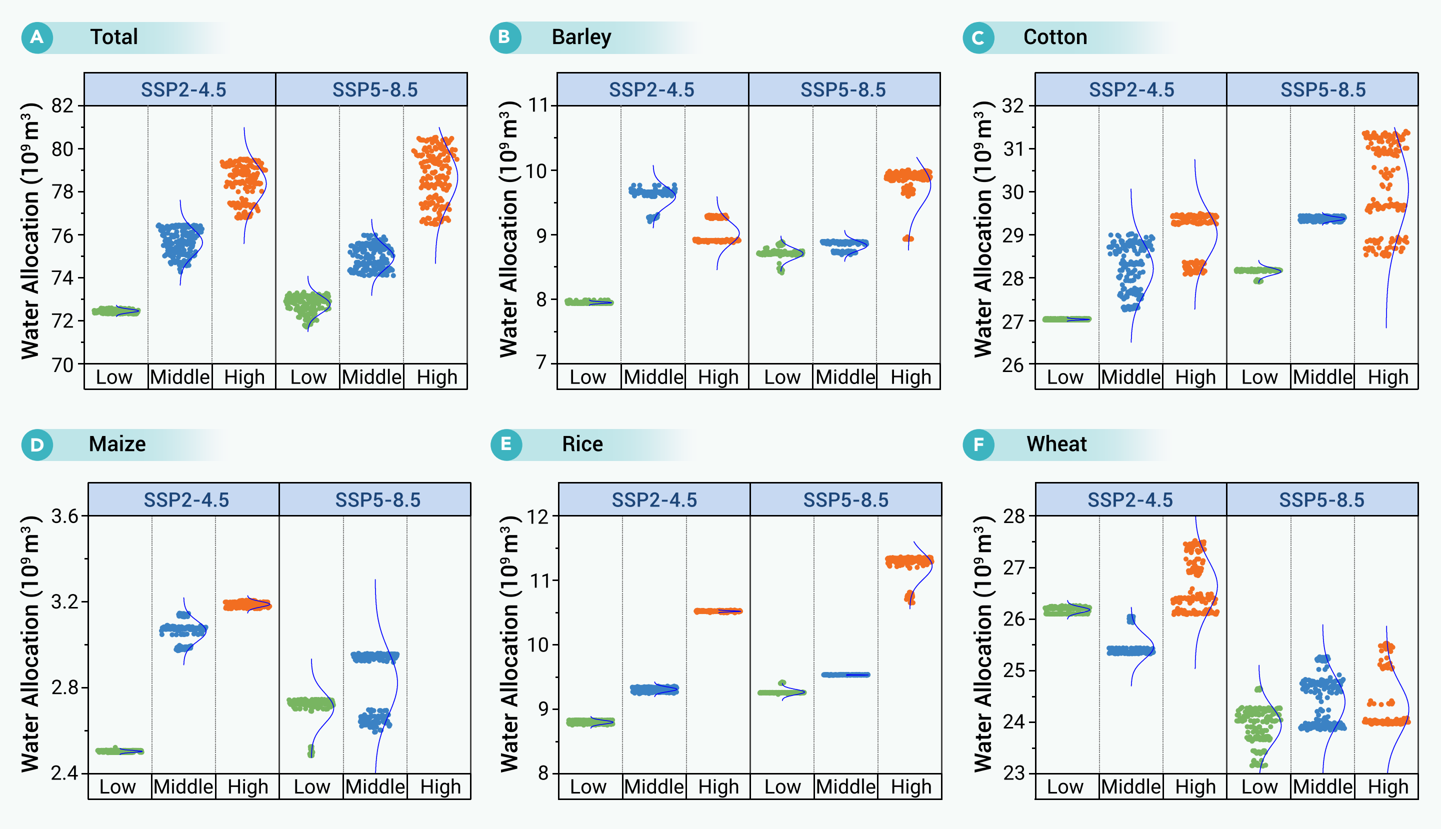

Figure 3. Crop water allocation under different SSPs and inflow scenarios

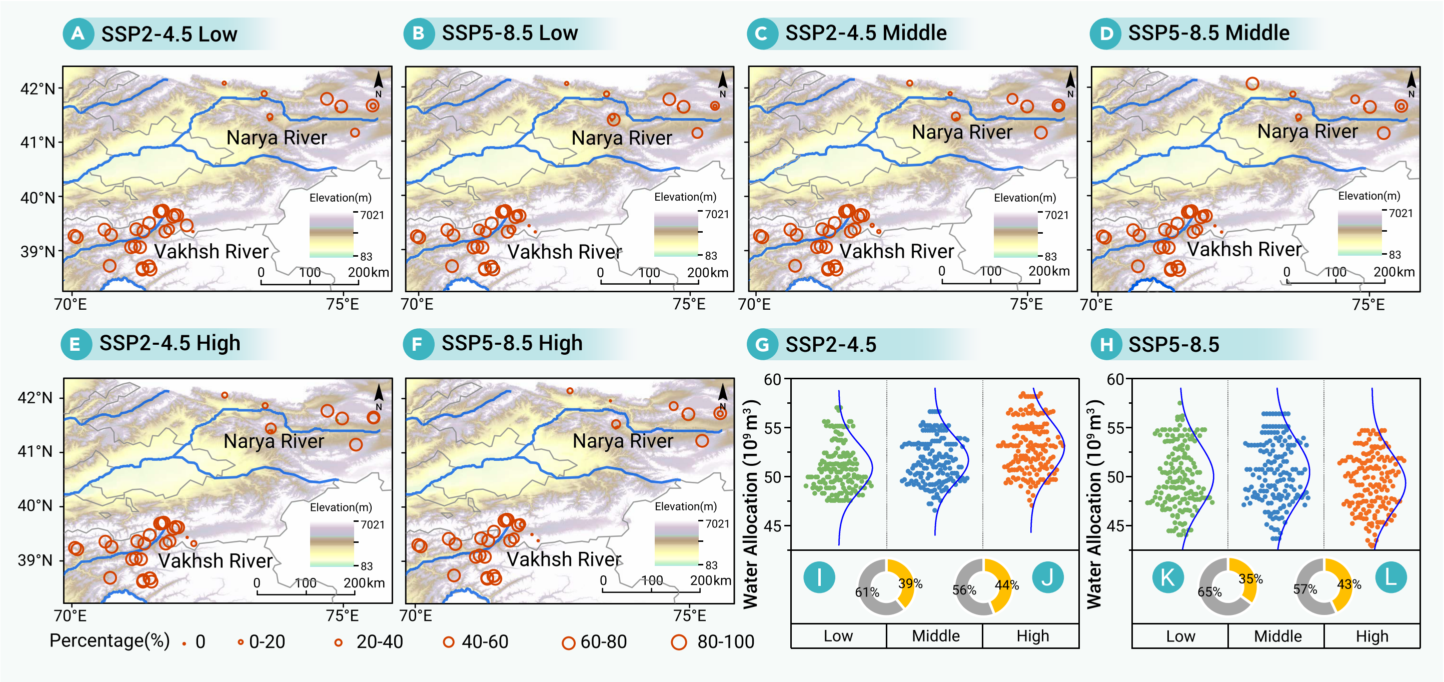

Figure 4. Hydropower water allocation under different SSPs and inflow scenarios Distribution of SPHS reservoirs and their probability of selection (A–F) and hydropower water allocation and the proportions of conventional reservoirs and SPHS reservoirs (G and H). The minimum (I and K) and maximum (J and L) proportions of SPHS reservoirs and conventional reservoirs under the SSP2-4.5 and SSP5-8.5 scenario. Standard map number GS (2025) 0904.

The integration of SPHS reservoirs significantly enhanced upstream controllable water resources, boosting them to a range of 42.91–58.47 × 10^9 m^3 across diverse climate and inflow scenarios (Figures 4G and 4H). A scenario-specific analysis revealed total resources of 46.55–58.47 × 10^9 m^3 under SSP2-4.5, compared with 42.91–57.49 × 10^9 m^3 under the more extreme SSP5-8.5. Critically, SPHS reservoirs constituted a substantial portion of this controllable supply. Under SSP5-8.5 scenarios, they provided 18.40–25.47 × 10^9 m^3, accounting for 35%–43% of the total, with conventional reservoirs supplying the remainder (Figures 4K and 4L). Their contribution under the SSP2-4.5 scenarios was proportionally higher at 39%–44% (14.95–24.82 × 10^9 m^3) (Figures 4I and 4J). However, this enhanced upstream capacity was partially offset by rising downstream agricultural and ecological water demands, a direct consequence of climate and socioeconomic factors under SSP5-8.5. These results suggest the pivotal regulatory function of SPHS systems in enhancing basin-wide water resilience, highlighting their importance as a key component of adaptive water resource management strategies in a climate-uncertain future.

SYSTEM ECONOMIC BENEFITS

The data revealed an influence of SSPs and inflow scenarios on projected system benefits (Figure 5). Under the SSP2-4.5 scenario, the benefits ranged from 6.07 to 6.51 × 10^9 US$ for low inflow, 6.28 to 6.76 × 10^9 US$ for medium inflow, and 6.37 to 6.93 × 10^9 US$ for high inflow (Figure 5A). Additionally, a broader distribution and significantly higher volatility were estimated under the SSP5-8.5 scenario, where benefits spanned 6.05–6.68 × 10^9, 5.98–6.78 × 10^9, and 6.15–7.01 × 10^9 US$ for the respective inflow (Figure 5B). Moreover, the impacts of different water inflow scenarios on system benefits showed significant differences between the SSP2-4.5 and SSP5-8.5 scenarios (Figures 5C and 5D). Further analysis highlighted a contrast in different sectors responding to SSPs and inflow conditions. Hydropower generation benefits remained stable, ranging from 3.41 to 3.91 × 10^9 and 3.33–4.10×10^9 US$ across all scenarios (SSP2-4.5 and SSP5-8.5). This stability indicates the strong regulatory capacity of the combined conventional and SPHS reservoirs, which potentially manage inflow variability to secure reliable energy production. In contrast, agricultural benefits were highly sensitive to different SSPs and inflow scenarios. Notably, under SSP2-4.5 scenarios, benefits ranged from 2.59 to 2.62 × 10^9 US$ (low inflow), 2.72–2.86 × 10^9 US$ (medium inflow), and 2.83–3.02 × 10^9 US$ (high inflow). Under SSP5-8.5, they ranged from 2.49 to 2.63 × 10^9, 2.55 to 2.69 × 10^9, and 2.62 to 2.99 × 10^9 US$ across their respective scenarios.

Figure 6 depicts the relationships between system benefits, water allocation equity, and GHG emissions. A significant positive correlation (R = 0.61–0.85) between system benefits and the Gini coefficient was evident, indicating a trade-off between them. As system benefits increased, water allocation equity diminished, reflecting the concentration of water use in the high-benefit perspective, particularly for agriculture and hydropower. This trade-off was stronger under the SSP5-8.5 scenarios (R = 0.78–0.85) compared with the SSP2-4.5 scenarios (R = 0.61–0.74), due to increased supply-demand imbalances under high-emission scenarios. Climate mitigation strategies could help reduce these disparities and enhance system coordination. The trade-off was also influenced by inflow conditions. High-inflow scenarios showed weaker correlations (0.61 for the SSP2-4.5 scenario, 0.78 for the SSP5-8.5 scenario) than the medium- and low-inflow scenarios (0.74 and 0.73 for the SSP2-4.5 scenario, 0.79 and 0.85 for the SSP5-8.5 scenario). This suggests that water scarcity amplifies the equity-efficiency trade-off.

Under different SSPs and inflow scenarios, the system benefits and GHG emissions also showed a positive correlation. This relationship was weak and statistically insignificant under the medium- and low-inflow scenarios, indicating a minimal impact of system benefits on GHG emissions. However, it becomes stronger and more significant under high-inflow scenarios (0.62 in the SSP2-4.5 scenario and 0.46 in the SSP5-8.5 scenario), primarily due to larger crop planting areas (Table 1) and higher agricultural GHG emissions. These results highlight the link between system benefits and environmental impacts, especially under high-inflow scenarios, where greater system benefits correspond to higher GHG emissions.

DISCUSSION

This study evaluated the operational roles of existing, under-construction, and planned conventional reservoirs, during both growing and non-growing seasons, and identified their optimal integration with SPHS reservoirs in the Aral Sea Basin. We developed the optimized water allocation strategies that balance water allocation equity, system benefits, and GHG emissions, while ensuring the restoration of the Aral Sea, food security, and reliable supply of clean hydropower across different SSPs and inflow scenarios.

Leveraging the complementary regulation of upstream and downstream reservoirs, coupled with the integration of multiple SPHS reservoirs, significantly

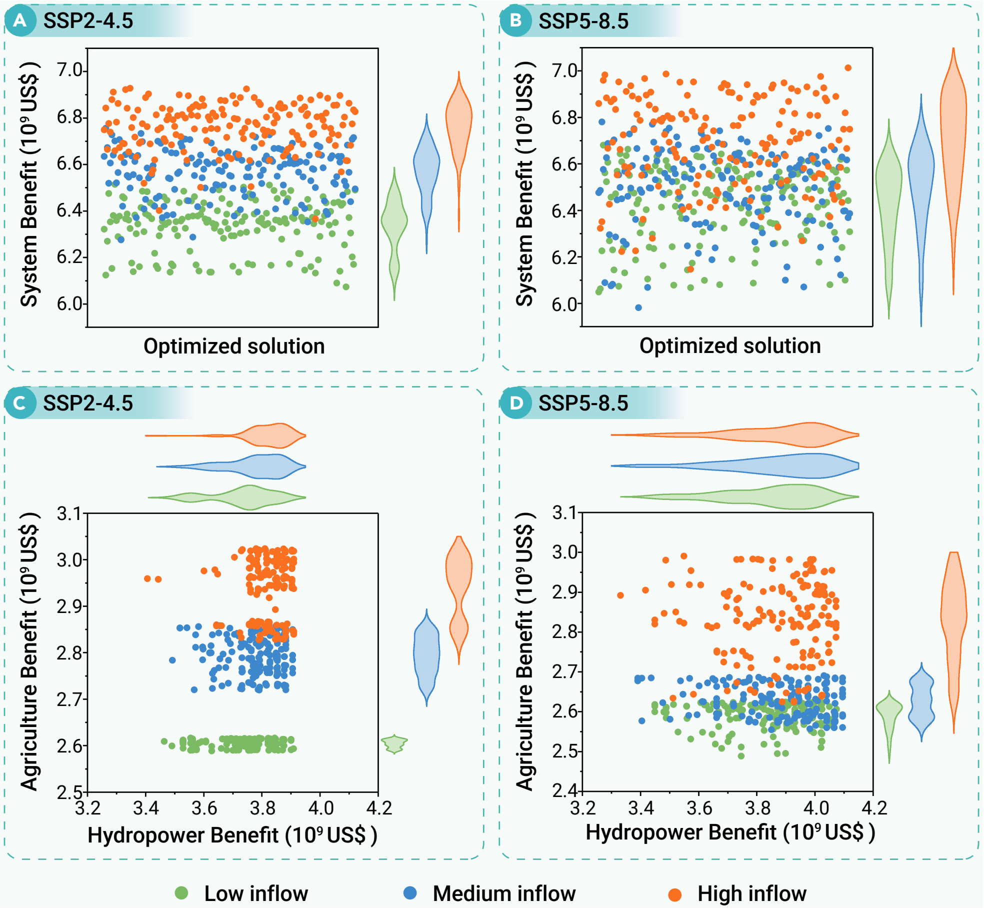

Figure 5. System, hydropower, and agricultural benefits under different SSPs and inflow scenarios The violin plots show the distribution of benefits under each inflow scenario, with the width of each plot indicating the density of values.

enhances water resources controllability across the Aral Sea Basin. The total volume of managed water resources available to meet agricultural, ecological, and hydropower energy demands reached 158.75–167.56 km^3 under varying scenarios (Figure 7). The increased controllability, particularly regarding water allocated for hydropower generation, played a key role in sustaining water allocation equity across all strategies (Figure 2). Therefore, the coordinated operations of conventional and SPHS reservoirs offers a critical mechanism for mitigating intersectoral water competition and provides a transferable reference for addressing similar challenges in stressed water resource system worldwide. For instance, Hunt et al.[49] identified India's Indus Basin as a cost-effective SPHS site with global storage potential, while de Assis Brasil Weber et al.[50] demonstrated the role of SPHS in mitigating the climate impacts on Brazil's power sector. Within the Aral Sea Basin, integrating SPHS reservoirs more effectively manages seasonal water variability and promotes sustainable water use. In addition, the regional agreement on the Kambar-Ata-1 reservoir and the strategic transitioning of the Toktogul to an irrigation reservoir reflect a growing institutional focus on equitable water resource allocation. This approach can also offer strategic insights for managing the major reservoir such as the Tajikistan's Nurek and Rogun reservoirs to optimize basin-wide coordination.

Furthermore, our analysis exhibited that projected ecological water volumes, ranging from about 35 to 38 $\times$ 10^9 m^3 across all scenarios (Figure 7), are sufficient to meet the ecological demands of the Aral Sea's restoration.[12] Concurrently, under the integrated application of flood and drip irrigation systems, agricultural water allocations are projected to average between 72.47 and 78.68 km^3 for all scenarios, supporting a cultivated area of around 5.2–5.6 million hectares for the five major crops. Under the more extreme SSP5-8.5 scenario, agricultural water use was projected between 72.76 and 78.68 km^3, sustaining a corresponding cultivated area of around 5.0–5.5 million hectares. This reallocation of water resources and the optimization of irrigation practices require a significant strategic reduction in total cultivated area. Compared with the current situations, these projected cultivated areas represented a substantial decrease of 14.37%–21.05% under the SSP2-4.5 and a more pronounced reduction of 16.16%–23.93% under the SSP5-8.5, indicating a critical trade-off between agriculture and ecological restoration. Similarly, Shi et al.[51] suggested that expanding drip irrigation could increase Aral Sea inflows by 6.4–9.6 km^3 annually. Likewise, coordinated management could increase ecological water allocation by 12.5% while reducing agricultural use by 17%,[16] a trend consistent with projections of agricultural declining water share.[17] A critical strategy involves reducing aggregated water allocation and sustaining food crops, such as wheat (29.1%–31.2% of the total area).[52] However, these restoration goals conflict with 8.2 million projections of irrigated area expansion by 2050, emphasizing critical need for policies that address ecological restoration and agricultural productivity.[53] Ultimately, sustainable resources use depends on fine-tuned water management and policy support. Promoting alternative, less-water-intensive economic sectors offers a viable strategy to reduce agricultural water reliance.

Water allocation exhibited a clear trade-off in the Aral Sea Basin between equity, system benefit, and GHG emissions, influenced by environmental factors. Crop structure adjustments illustrate this tension, as high-input crops can generate higher revenues but also increase GHG emissions, thereby reducing the carbon efficiency of agricultural system. Moreover, if poorly balanced, they may further exacerbate soil salinization and degradation, undermining long-term food security and economic resilience.[51] This trade-off is also reflected in existing evidence on hydropower production, as reservoir regulation, while alleviating regional water competition and increasing hydropower output, may generate methane emissions during impoundment and discharge, offsetting part of its low-carbon advantage, with global emissions estimated at about 0.38 Pg CO_2eq year^{-1}.[54] Although such reservoir-related emissions were not included within the scope of this study due to data limitations, they represent an additional factor that could further intensify the trade-off. Taken together, these highlight that the water-food-energy-environment nexus cannot be effectively managed through sectoral strategies alone but requires integrated, cross-sectoral approaches. Ma et al.[19] emphasized that sustainable development in Central Asia requires balancing system risks and benefits, which may sacrifice some ecological gains. Therefore, integrated approaches achieve trade-offs and improve resource efficiency more effectively than one single sector strategies. Qin et al.[15] noted that food trade amplifies nexus pressures, linking regional resource to global economic and ecological changes and increasing management complexity. This underscores the need for regional cooperation and transboundary coordination. Notably, integrated management strategies require not only scientifically sound water allocation optimization but, more importantly, build consensus and cooperation among stakeholders. Besides, the political, economic, and interest structures are highly complex, with persistent strategic bilateral friction among Central Asian countries. Although optimization models can theoretically propose positive solutions, their efficacy is constrained by inadequate collaboration. For instance, the International Fund for Saving the Aral Sea (IFAS) and the Interstate Commission on Sustainable Development (ICSD) are weakened by limited information exchange and competing national priorities, resulting in unstable institutional arrangements.[4] To be effective, integrated basin management requires an adequate definition and implementation strategy. Fundamentally, adopting a

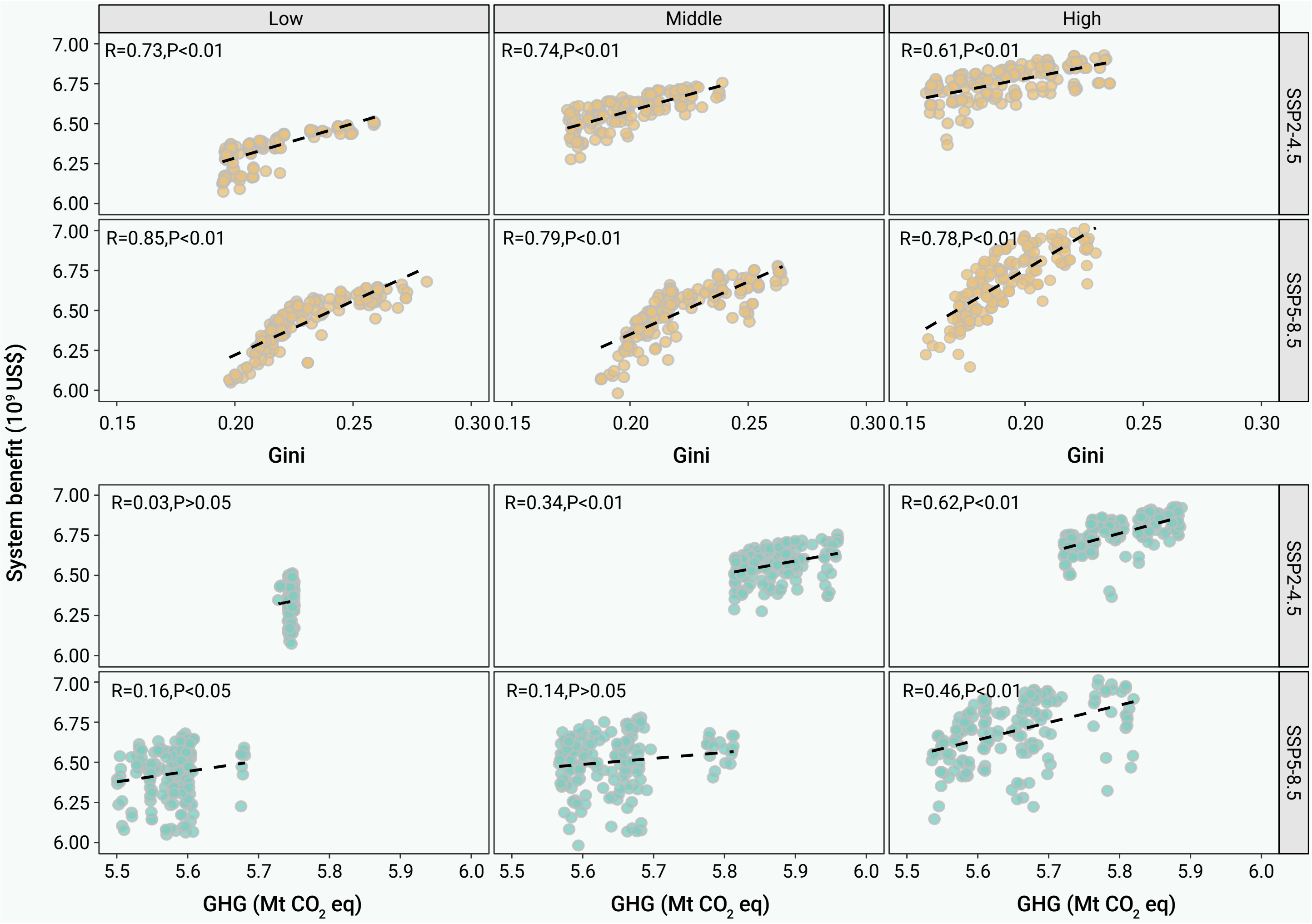

Figure 6. Relationship between system benefit, water allocation equity, and GHG emission under different SSPs and inflow scenarios

basin-wide perspective to transcend sectoral and national silos, coordinating water, energy, food, and ecological objectives. Key implementation pathways involve strengthening the mandate of existing institutions such as the IFAS, and ICSD; establishing regular dialogue and data-sharing platforms for hydrological monitoring and reservoir operations; embedding multi-objective optimization models into policymaking to inform negotiations; and designing equitable benefit-sharing mechanisms. Together, these measures can alleviate water competition, enhance the joint resource security, and provide the institutional foundations for long-term sustainable development of the basin.

LIMITATIONS AND UNCERTAINTIES

While this study provides an innovative framework to mitigate competition for water among agricultural, hydropower, and ecological sectors in the Aral Sea Basin, some limitations exist. Firstly, the agricultural analysis included limited data, omitting inputs such as seeds and machinery to focus solely on irrigation and fertilizers. Besides, the quantification of hydropower GHG emissions was limited by lack of regional data; and the absence of site-specific measurements for major reservoirs (e.g., Toktogul and Nurek) means applying global intensity bands without regional validation could bias the results. Previous analyses suggested that reservoir emissions, especially methane from anaerobic decay, can be substantial, particularly in younger, nutrient-rich systems or those with strong water-level changes, as seen in arid regions like the Aral Sea Basin.[55,56] The omission of these emissions implies that the net GHG trade-offs presented here should be interpreted as conservative, thus future assessment would benefit from monitoring and process-based reservoir carbon-flux. Additionally, the performance of under-construction and planned reservoirs may deviate from their design parameters due to hydrological variability, construction inaccuracies, or unexpected structural issues, introducing uncertainties into projections. Some critical parameters, including crop prices, hydropower tariffs, and fertilizer application rates, were derived from current data due to the absence of reliable long-term projections. Lastly, the indicators for water resource allocation were based on per capita controllable water resources. While this single metric captures certain aspects of fairness, it does not fully reflect the multidimensional influences of socioeconomic and ecological conditions. Overall, these assumptions and data limitations introduce uncertainties and may underestimate the complexity of the water-food-energy-environment nexus in the Aral Sea Basin. Therefore, future studies should consider enriching socioeconomic and ecological datasets, incorporating multidimensional allocation indicators, and validating reservoir operations with empirical data. Such efforts are essential for model calibration, enhancing the simulation capability, and improve the practical policy relevance of water resources optimization frameworks.

In addition to the parameter-related limitations discussed above, the robustness of the optimization outcomes was systematically evaluated through a scenario-based sensitivity analysis of the solution, with the solution being computed using independent runs to enhance its diversity and stability. Using mean-based, range-based indices, and the two-way analysis of variance (ANOVA), we assessed the sensitivity to flow variability, SSPs, and their interaction (supplemental information Text S3). The results demonstrate general robustness, as no objectives were highly sensitive across all scenarios (Figure S2). However, sectoral sensitivities differed, where agriculture benefits were more sensitive to flow variability due to water dependencies, while hydropower benefits and GHG emissions were mainly shaped by SSPs, through their influence on long-term energy demand and development trajectories. Equity (Gini coefficient) was influenced by both factors. It deteriorated under low-flow conditions but also responded to differences in SSPs that alter water

www.the-innovation.org

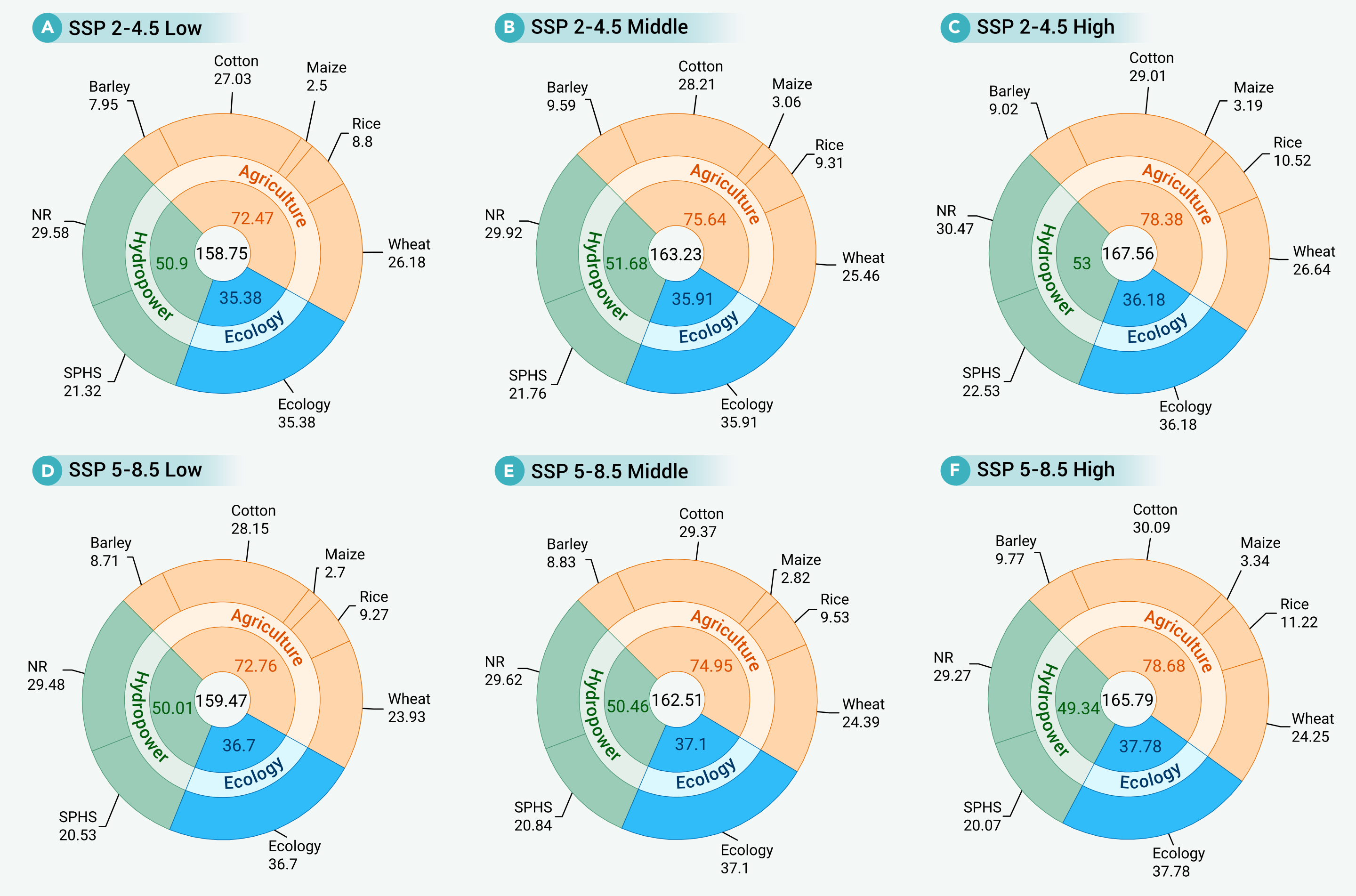

Figure 7. Average water allocation for agriculture, hydropower, and ecology under different SSPs and inflow scenarios Note: shown are the average water allocation for agriculture, hydropower, and ecology based on all multi-objective optimization outcomes under different SSPs and inflow scenarios. Unit: 10^9 m^3.

allocation priorities. ANOVA confirmed that flow explains most agricultural benefits variance and SSPs account for most variance in hydropower benefits and GHG emission, with negligible interaction effects. These findings provide strong evidence that the main conclusions of this study remain stable across a wide range of plausible future conditions, and they highlight the need to prioritize hydrological uncertainties for agriculture and socioeconomic trajectories for energy and environmental management.

CONCLUSION

This study investigates the seasonal roles and optimal integration of conventional and with SPHS reservoirs, employing the NSGA-III algorithm to develop water allocation strategies that balance equity, system benefits, and GHG emissions while supporting ecological restoration, food security, and clean energy provision. The following were the main findings.

- Gini coefficients remained below 0.29 across all scenarios, indicating high equity alongside balanced economic and ecological outcomes.
- Agricultural water use ranged from 71.71 × 10^9 to 80.53 × 10^9 m^3 across all scenarios, corresponding to crop area of 5,160.32 × 10^3 to 5,595.79 × 10^3 ha under SSP2-4.5 and 4,972.09 × 10^3 to 5,480.07 × 10^3 ha under SSP5-8.5, representing reductions of 14.37%–21.05% and 16.16%–23.93%, respectively, relative to current levels. SPHS reservoirs increased upstream controllable water for hydropower to 42.91–58.47 × 10^9 m^3, contributing 35%–44% of the total. Besides, hydropower output remained stable due to coordination between conventional and SPHS reservoirs. In addition, the water allocated to the Aral Sea was sufficient to satisfy ecological restoration requirements, amounting to 35.38–36.18 × 10^9 m^3 under SSP2-4.5 and 36.70–37.78 × 10^9 m^3 under SSP5-8.5.
- System benefits exhibited higher variability under SSP5-8.5, suggesting sensitivity to external pressures. Notably, a pronounced trade-off was observed between system benefits and allocation equity (R = 0.61–0.85), particularly under high-emission and low-inflow scenarios, while system benefits were positively associated with GHG emissions (R = 0.03–0.62).

These results highlighted the critical need for integrated water management to address the interconnected challenges of water, food, energy, and environment sustainability in the Aral Sea Basin.

RESOURCE AVAILABILITY

Materials availability

This study did not generate new unique materials or reagents.

Data and code availability

- All data used in this study are publicly available and have been described in detail in the materials and methods.
- No new observational data were created for this research.
- The multi-objective optimization analyses were conducted in MATLAB using the publicly available PlatEMO framework.
- All Pareto solution sets reported in this study are available from the corresponding author upon reasonable request; no custom codes beyond the standard PlatEMO environment were developed.

FUNDING AND ACKNOWLEDGMENTS

The work was jointly supported by the Key Research Task of the CAS Research Center for Ecology and Environment of Central Asia (grant no. 1117007001), the Strategic Priority Research Program of the Chinese Academy of Sciences (grant no.

XDB0720400), the National Youth Talent Project (grant no. E4150103), and the Science Foundation of Xinjiang Institute of Ecology and Geography, Chinese Academy of Sciences (grant no. E3508501). Y.H. was supported by a grant from the program of China Scholarship Council (ICPIT–International Cooperative Program for Innovative Talents, grant no. 202310630004) during her stay in Ghent University, Ghent, Belgium. The funders had no role in study design, data collection and analysis, decision to publish, or preparation of the manuscript.

AUTHOR CONTRIBUTIONS

Methodology, Y.H., Y.D., and W.W.; writing – original draft, Y.H.; writing – review & editing, W.D., S.Z., P.M.K., P.D.M., and P.L.M.G.; supervision, G.S., W.D., S.Z., and Y.C.; conceptualization, W.D.

DECLARATION OF INTERESTS

The authors declare no competing interests.

SUPPLEMENTAL INFORMATION

It can be found online at https://doi.org/10.1016/j.xinn.2025.101177.

REFERENCES

1. Boretti, A. and Rosa, L. (2019). Reassessing the projections of the World Water Development Report. *NPJ Clean Water* **2**:15. DOI:10.1038/s41545-019-0039-9
2. Zhou, Y.X., Li, Y.P., Huang, G.H. et al. (2022). A fixed-mix stochastic fractional programming method for optimizing agricultural irrigation and hydropower generation in Central Asia. *J. Contam. Hydrol.* **248**:104004. DOI:10.1016/j.jconhyd.2022.104004
3. Chen, Y., Li, Z., Fang, G. et al. (2018). Large Hydrological Processes Changes in the Transboundary Rivers of Central Asia. *JGR. Atmospheres* **123**:5059–5069. DOI:10.1029/2017jd028184
4. Wang, X., Chen, Y., Li, Z. et al. (2021). Water resources management and dynamic changes in water politics in the transboundary river basins of Central Asia. *Hydrol. Earth Syst. Sci.* **25**:3281–3299. DOI:10.5194/hess-25-3281-2021
5. Crétaux, J.-F., Biancamaria, S., Arsen, A. et al. (2015). Global surveys of reservoirs and lakes from satellites and regional application to the Syrdarya river basin. *Environ. Res. Lett.* **10**:015002. DOI:10.1088/1748-9326/10/1/015002
6. Jalilov, S.-M., Keskinen, M., Varis, O. et al. (2016). Managing the water–energy–food nexus: Gains and losses from new water development in Amu Darya River Basin. *J. Hydrol. X.* **539**:648–661. DOI:10.1016/j.jhydrol.2016.05.071
7. Ahmadzai, S. and McKinna, A. (2018). Afghanistan electrical energy and trans-boundary water systems analyses: Challenges and opportunities. *Energy Rep.* **4**:435–469. DOI:10.1016/j.egyr.2018.06.003
8. Wang, Y., Luo, G., Li, C. et al. (2022). Net carbon flux from cropland changes in the Central Asian Aral Sea Basin. *J. Environ. Manage.* **314**:115078. DOI:10.1016/j.jenvman.2022.115078
9. Yang, X., Wang, N., Chen, A. et al. (2020). Changes in area and water volume of the Aral Sea in the arid Central Asia over the period of 1960–2018 and their causes. *Catena* **191**:104566. DOI:10.1016/j.catena.2020.104566
10. Micklin, P. (2016). The future Aral Sea: hope and despair. *Environ. Earth Sci.* **75**:844. DOI:10.1007/s12665-016-5614-5
11. Hu, Z., Zhang, C., Hu, Q. et al. (2014). Temperature Changes in Central Asia from 1979 to 2011 Based on Multiple Datasets. *J. Clim.* **27**:1143–1167. DOI:10.1175/jcli-d-13-00064.1
12. Wang, X., Zhang, J., Wang, S. et al. (2023). Reviving the Aral Sea: A Hydro-Eco-Social Perspective. *Earths Future* **11**:e2023EF003657. DOI:10.1029/2023ef003657
13. White, C.J., Tanton, T.W. and Rycroft, D.W. (2014). The Impact of Climate Change on the Water Resources of the Amu Darya Basin in Central Asia. *Water Resour. Manage.* **28**:5267–5281. DOI:10.1007/s11269-014-0716-x
14. Rakhmatullaev, S. and Abdullaev, I. (2014). Central Asian irrigation sector in a climate change context: some reflections. *J. Water Clim. Chang.* **5**:341–356. DOI:10.2166/wcc.2014.120
15. Qin, J., Duan, W., Chen, Y. et al. (2022). Comprehensive evaluation and sustainable development of water–energy–food–ecology systems in Central Asia. *Renew. Sustain. Energy Rev.* **157**:112061. DOI:10.1016/j.rser.2021.112061
16. Ma, Y., Li, Y.P., Zhang, Y.F. et al. (2021). Mathematical modeling for planning water-food-ecology-energy nexus system under uncertainty: A case study of the Aral Sea Basin. *J. Clean. Prod.* **308**:127368. DOI:10.1016/j.jclepro.2021.127368
17. Zhang, Y.F., Li, Y.P., Huang, G.H. et al. (2021). A copula-based stochastic fractional programming method for optimizing water-food-energy nexus system under uncertainty in the Aral Sea basin. *J. Clean. Prod.* **292**:126037. DOI:10.1016/j.jclepro.2021.126037
18. Ruan, H., Yu, J., Wang, P. et al. (2023). Relieving water stress by optimizing crop structure is a practicable approach in arid transboundary rivers of Central Asia. *Agric. Water Manag.* **275**:108055. DOI:10.1016/j.agwat.2022.108055
19. Ma, Y., Li, Y., Wang, H. et al. (2024). Non-deterministic multi-level model for planning water-ecology nexus system under climate change. *Cell Rep. Sustain.* **1**:100032. DOI:10.1016/j.crsus.2024.100032
20. Hunt, J.D., Byers, E., Wada, Y. et al. (2020). Global resource potential of seasonal pumped hydropower storage for energy and water storage. *Nat. Commun.* **11**:947. DOI:10.1038/s41467-020-14555-y
21. Zakeri, B., Hunt, J.D., Laldjebaev, M. et al. (2022). Role of energy storage in energy and water security in Central Asia. *J. Energy Storage* **50**:104587. DOI:10.1016/j.est.2022.104587
22. Marti, B., Yakovlev, A., Karger, D.N. et al. (2023). CA-discharge: Geo-Located Discharge Time Series for Mountainous Rivers in Central Asia. *Sci. Data* **10**:579. DOI:10.1038/s41597-023-02474-8
23. Gulakhmadov, A., Chen, X., Gulakhmadov, M. et al. (2021). Evaluation of the CRU TS3.1, APHRODITE_V1101, and CFSR Datasets in Assessing Water Balance Components in the Upper Vakhsh River Basin in Central Asia. *Atmosphere* **12**:1334. DOI:10.3390/atmos12101334
24. Shang, H. (2019). Monthly streamflow of Panj River in the Amu Dyrya catchment in Central Asia (1967-2017). *A Big Earth Data Platform for Three Poles*
25. Wei, W., Zou, S., Duan, W. et al. (2024). Increased population exposures to extreme precipitation in Central Asia under 1.5 °C and 2 °C global warming scenarios. *Geogr. Sustainability* **5**:343–356. DOI:10.1016/j.geosus.2024.02.005
26. Bjarke, N., Barsugli, J. and Livneh, B. (2023). Ensemble of CMIP6 derived reference and potential evapotranspiration with radiative and advective components. *Sci. Data* **10**:417. DOI:10.1038/s41597-023-02290-0
27. Frolking, S., Wisser, D., Grogan, D. et al. (2020). GAEZ+_2015 Crop Harvest Area, V4 ed. (Harvard Dataverse)
28. Khan, Z., Thompson, I., Vernon, C. et al. (2022). Output Data: tethys_v1.3.1_main_ssp_rcp. V1 ed. *Harvard Dataverse*
29. van Dijk, M., Morley, T., Rau, M.L. et al. (2021). A meta-analysis of projected global food demand and population at risk of hunger for the period 2010–2050. *Nat. Food* **2**:494–501. DOI:10.1038/s43016-021-00322-9
30. Brinkerink, M., Gallachóir, B.Ó. and Deane, P. (2021). Building and Calibrating a Country-Level Detailed Global Electricity Model Based on Public Data. *Energy Strategy Rev.* **33**:100592. DOI:10.1016/j.esr.2020.100592
31. Wichelns, D., Anarbekov, O., Jumaboev, K. et al. (2010). Irrigation Pricing Alternatives for Water User Associations in Central Asia. In IWMI Conference Proceedings 211317 (International Water Management Institute)
32. He, S., Foley, A., Chen, N. et al. (2021). Cooperative electric energy transmission between Central Asia and Pakistan. *Energy Strategy Rev.* **38**:100756. DOI:10.1016/j.esr.2021.100756
33. Asian Development Bank (2013). Access to Green Finance Project (Project No. 45229-001) (Asian Development Bank). https://www.adb.org/projects/45229-001/main
34. Ray, D.K., Sloat, L.L., Garcia, A.S. et al. (2022). Crop harvests for direct food use insufficient to meet the UN's food security goal. *Nat. Food* **3**:367–374. DOI:10.1038/s43016-022-00504-z
35. Akhmedov, K.M.A. and Petrov, G. (2019). The calculation of the cost of water in the regulation of river runoff. In International Conference on Innovative Applied Energy in Oxford City, United Kingdom. DOI:10.13140/RG.2.2.16964.99200
36. Normatov, I. (2017). Risk management and assessment of water resources of the Amudarya River Basin under condition of climate change and construction of large reservoirs. In The Implementation Stages, Results and Recommendations of the PEER Project 4-356
37. Liang, W., Chen, Y., Fang, G. et al. (2023). Machine learning method is an alternative for the hydrological model in an alpine catchment in the Tianshan region, Central Asia. *J. Hydrol.: Reg. Stud.* **49**:101492. DOI:10.1016/j.ejrh.2023.101492
38. Huang, H., Liang, Z., Li, B. et al. (2019). Combination of Multiple Data-Driven Models for Long-Term Monthly Runoff Predictions Based on Bayesian Model Averaging. *Water Resour. Manage.* **33**:3321–3338. DOI:10.1007/s11269-019-02305-9
39. Wang, X., Chen, Y., Li, Z. et al. (2020). The impact of climate change and human activities on the Aral Sea Basin over the past 50 years. *Atmos. Res.* **245**:105125. DOI:10.1016/j.atmosres.2020.105125
40. Deb, K. and Jain, H. (2014). An Evolutionary Many-Objective Optimization Algorithm Using Reference-Point-Based Nondominated Sorting Approach, Part I: Solving Problems With Box Constraints. *IEEE Trans. Evol. Comput.* **18**:577–601. DOI:10.1109/tevc.2013.2281535
41. Chen, Y., Wang, M., Zhang, Y. et al. (2023). Cascade Hydropower System Operation Considering Ecological Flow Based on Different Multi-Objective Genetic Algorithms. *Water Resour. Manage.* **37**:3093–3110. DOI:10.1007/s11269-023-03491-3
42. Zhiltsov, S.S. (2024). Central Asia: Region's Potential and New Challenges. *RUDN Journal of Political Science* **26**:78–89. DOI:10.22363/2313-1438-2024-26-1-78-89
43. Wegerich, K., Van Rooijen, D., Soliev, I. et al. (2015). Water Security in the Syr Darya Basin. *Water* **7**:4657–4684. DOI:10.3390/w7094657
44. Zhou, M., Sun, D., Wang, X. et al. (2024). Multi-objective optimal allocation of water resources in Shule River Basin of Northwest China based on climate change scenarios. *Agric. Water Manag.* **302**:109015. DOI:10.1016/j.agwat.2024.109015
45. Su, F., Pritchard, H.D., Yao, T. et al. (2022). Contrasting Fate of Western Third Pole's Water Resources Under 21st Century Climate Change. *Earths Future* **10**:e2022EF002776. DOI:10.1029/2022ef002776
46. Nunez, S., Alkemade, R., Kok, K. et al. (2020). Potential biodiversity change in Central Asian grasslands: scenarios for the impact of climate and land-use change. *Reg. Environ. Change* **20**:39. DOI:10.1007/s10113-020-01619-4
47. Vollset, S.E., Goren, E., Yuan, C.-W. et al. (2020). Fertility, mortality, migration, and population scenarios for 195 countries and territories from 2017 to 2100: a forecasting analysis

for the Global Burden of Disease Study. *Lancet* **396**:1285–1306. DOI:10.1016/S0140-6736(20)30677-2

48. Renard, D. and Tilman, D. (2019). National food production stabilized by crop diversity. *Nature* **571**:257–260. DOI:10.1038/s41586-019-1316-y
49. Hunt, J.D., Falchetta, G., Parkinson, S. et al. (2021). Hydropower and seasonal pumped hydropower storage in the Indus basin:pros and cons. *J. Energy Storage* **41**:102916. DOI:10.1016/j.est.2021.102916
50. de Assis Brasil Weber, N., Hunt, J.D., Zakeri, B. et al. (2024). Seasonal pumped hydropower storage role in responding to climate change impacts on the Brazilian electrical sector. *J. Energy Storage* **87**:111249. DOI:10.1016/j.est.2024.111249
51. Shi, H., Luo, G., Zheng, H. et al. (2020). Coupling the water-energy-food-ecology nexus into a Bayesian network for water resources analysis and management in the Syr Darya River basin. *J. Hydrol. X.* **581**:124387. DOI:10.1016/j.jhydrol.2019.124387
52. Ma, Y., Li, Y.P., Huang, G.H. et al. (2023). Sustainable management of water-agriculture-ecology nexus system under multiple uncertainties. *J. Environ. Manage.* **341**:118096. DOI:10.1016/j.jenvman.2023.118096
53. Narbayep, M. and Pavlova, V. (2022). The Aral Sea, Central Asian Countries and Climate Change in the 21st Century (United Nations ESCAP)
54. Li, M. and He, N. (2022). Carbon intensity of global existing and future hydropower reservoirs. *Renew. Sustain. Energy Rev.* **162**:112433. DOI:10.1016/j.rser.2022.112433
55. Keller, P.S., Marcé, R., Obrador, B. et al. (2021). Global carbon budget of reservoirs is overturned by the quantification of drawdown areas. *Nat. Geosci.* **14**:402–408. DOI:10.1038/s41561-021-00734-z
56. Soued, C., Harrison, J.A., Mercier-Blais, S. et al. (2022). Reservoir CO2 and CH4 emissions and their climate impact over the period 1900–2060. *Nat. Geosci.* **15**:700–705. DOI:10.1038/s41561-022-01004-2

The Innovation, Volume 7

Supplemental Information

A transboundary water allocation strategy for the Aral Sea Basin: Integrating the water-food-energy-environment nexus

Yanan Hu, Guangdong Sun, Weili Duan, Shan Zou, Yanfeng Di, Yaning Chen, Patient Mindje Kayumba, Wei Wei, Philippe De Maeyer, and Peter L.M. Goethals

TEXT S1:

The Random Forest process involves[1]: (1) generating bootstrap samples from the training set (N samples) to construct individual decision trees; (2) randomly selecting m features (m ≪ M) from M input features at each node split and choosing the optimal split point, a key mechanism to reduce overfitting and enhance generalization; and (3) allowing each tree to grow fully without pruning. The final prediction is obtained by averaging the outputs of all trees, balancing individual tree complexity with ensemble generalization error control.

The Random Forest model was assessed using an out-of-sample validation strategy. Specially, 80% of the historical runoff and hydrometeorological data were employed for model calibration, while the remaining 20% were withheld as an independent validation set. This approach is widely recognized in hydrological modelling as a reliable means to evaluate predictive skill and to minimize the risk of overfitting. Model performance, shown in Fig. S1, indicates strong predictive capability, with Coefficient of Determination (R^2) values of 0.72–0.90 and Nash-Sutcliffe efficiency coefficient (NSE) of 0.91–0.96 for the three stations. These results demonstrate that the Random Forest model captures the nonlinear relationships between predictors and runoff and provides robust and reliable out-of-sample predictions.

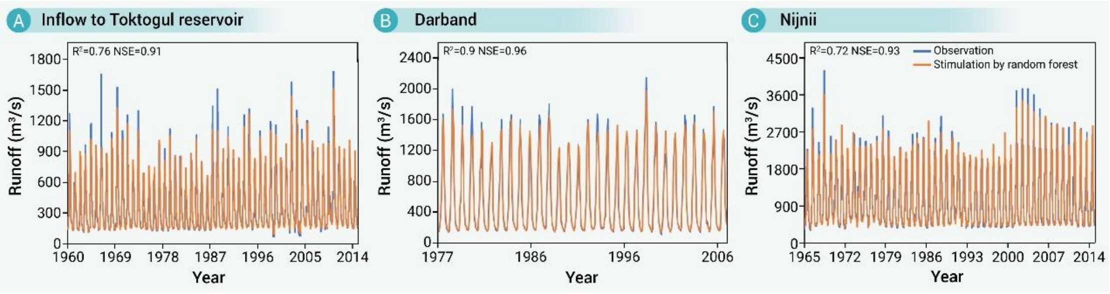

Fig. S1. Comparison and validation of observed and simulated runoff in the Aral Sea Basin

TEXT S2:

The CROPWAT model was used to calculate the irrigation water requirements for five crops. CROPWAT, a tool based on the Penman-Monteith formula, estimates crop water needs and irrigation demand. The equations are as follows:

$$ET_c = K_c * ET_0 \quad (1)$$

Where ET_c is the crop water demand,，K_c is the crop coefficient for different growth stages (parameters for the five Central Asian crops were taken from Liu et al.[2])，and ET_0 the potential evapotranspiration.

$$P_e = P * \frac{4.17 - 0.2 * P}{4.17}, for\ P < 8.3mm/day \quad (2)$$

$$P_e = 4.17 + 0.1 * \mathrm{P}, \mathrm{for\ P} \geq 8.3\mathrm{mm/day} \quad (3)$$

where Pe is effective precipitation and P is the precipitation.

$$CIR = Area * 10 * (ET_0 - P_e)/I \quad (4)$$

where CIR represents the crop irrigation requirement，Area is the crop area，and the constant 10 converts water depth (mm) into water volume (m^3/ha)，I is the irrigation efficiency, which in this study considers a combination of flood and drip irrigation with an efficiency of 50%, based on coefficients from Wang et al.[3].

TEXT S3:

To evaluate the robustness of the optimization results under uncertain future conditions, two primary sources of uncertainty were considered: socioeconomic shared pathways and hydrological inflow variability. For each optimization objective, sensitivity was assessed using both mean-based and range-based indicators.[4] Mean-based sensitivity measures the average deviation of objectives under different SSPs and Flow conditions, reflecting the influence of uncertainty on overall trends. Range-based sensitivity captures the difference between maximum and minimum values across scenarios, emphasizing system performance under extreme conditions. Furthermore, a two-way analysis of variance (ANOVA) was applied to partition the outcome variance into contributions from SSP, Flow, and their interaction.[5] The effect size, quantified by η^2 (eta squared), indicates the proportion of variance explained by each factor, thereby identifying the dominant drivers and interaction effects. By integrating mean-based sensitivity, range-based sensitivity, and variance decomposition, this framework provides a comprehensive characterization of how uncertainties propagate into equity, hydropower, agricultural, and environmental objectives, ensuring that comparative evaluations of alternative water allocation strategies remain robust across scenarios.

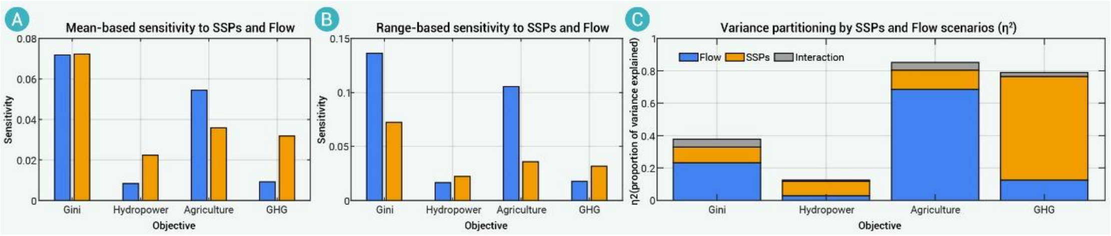

Fig. S2. Sensitivity analysis and ANOVA under SSPs and flow scenarios

Table S1. Details of the CMIP6 global climate models used in this study

Number	Model	Institute	Country
1	CanESM5	Canadian Centre for Climate Modelling and Analysis	Canada
2	CMCC-CM2-SR5	Fondazione Centro Euro-Mediterraneo sui Cambiamenti Climatici	Italy
3	CMCC-ESM2	Fondazione Centro Euro-Mediterraneo sui Cambiamenti Climatici	Italy
4	GFDL-ESM4	NOAA Geophysical Fluid Dynamics Laboratory	USA
5	INM-CM4-8	Institute for Numerical Mathematics	Russia
6	INM-CM5-0	Institute for Numerical Mathematics	Russia
7	IPSL-CM6A-LR	Institute Pierre-Simon Laplace	France
8	MIROC6	Institute of Atmospheric Oceanography, Japan Agency of Marine Earth Science and Technology	Japan
9	NorESM2-LM	Norwegian Climate Centre	Norway
10	NorESM2-MM	Norwegian Climate Centre	Norway
11	TaiESM1	Research Center for Environmental Changes, Academia Sinica	Taiwan, China

Table S2. Nomenclature for parameters and variables.

Indices	Definition
I	Country
J	Reservoir
K	Seasonal pumped hydropower storage reservoir
T	Crop type
X	Crop growing season and non-growing season
PO_i	Population of country i in the basin
W_i	The amount of water that can control in country i (hydropower in upstream countries, agriculture in downstream countries) (m^3)
HW_j	Electricity generation per unit water of reservoir j (kwh/m^3)
WA_{jh}	water available for hydropower of reservoir j (m^3)
HEC_i	Household electricity consumption of country i (kWh)
E_k	Seasonal pumped hydropower storage electricity generation (kWh)
P_{hi}	Household electricity prices in country i (US$/kWh)
P_{bi}	Business electricity prices in country i (US$/kWh)
C_k	total cost of seasonal pumped storage reservoir k (US$)
A	Total crop area in the Aral Sea basin (ha)
A_{it}	Area of crop t in country i (ha)
Y_{it}	Yield per unit area of crop t in country i (ton/ha)
P_{it}	Price of crop t in country i (US$/ton)
P_{it}^{irri}	Irrigation cost per unit area of crop t in country i (US$/ha)
P_{it}^{fert}	Fertilizer cost per unit area of crop t in country i (US$/ha)
N_i	Nitrogen fertilizer application per unit area in country i (kg/ha)
EF_d	Direct emission factor for N fertilizer application
F_{vol}	Fraction of the ammonia volatilization
EF_{vol}	Emission factor of ammonia volatilization
F_{leach}	Fraction of the nitrogen leaching losses
EF_{leach}	Emission factor of nitrogen leaching
A_i^{rice}	Rice harvested area in country i (ha)
EF_{CH_4}	Emission factor of rice
WD_t	Water demand per unit area of crop t (m^3/ha)
WS_e	Ecological water supply for the Aral Sea (m^3)
WD_e	Ecological water demand of the Aral Sea (m^3)
TAW	Total available water (m^3)
WD_{io}	Industrial, municipal and livestock water demand in country i (m^3)
SAW	Seasonal water availability (m^3)
FD	Per capita food demand (ton/per capita)
V	Reservoir water storage (m^3)
V_{min}	Minimum water storage capacity of the reservoir (m^3)
V_{max}	Maximum water storage capacity of the reservoir (m^3)
HD	Hydropower demand in the basin (kWh)

Table S3. The probability of selecting seasonal pumped hydropower storage reservoirs

					SSP2-4.5			SSP5-8.5	
ID	FID	Lat	Lon	Low	Middle	High	Low	Middle	High
1	172	42.05	72.82	26.06%	14.55%	32.73%	6.67%	100.00%	32.12%
2	173	41.87	73.56	20.00%	23.64%	31.52%	24.24%	23.03%	0.00%
3	168	41.45	73.67	51.52%	40.00%	63.03%	35.76%	37.58%	41.82%
4	179	41.38	73.65	0.00%	0.00%	20.00%	0.00%	0.00%	0.00%
5	186	41.40	73.69	0.00%	0.00%	0.00%	100.00%	0.00%	0.00%
6	166	41.77	74.71	69.70%	85.45%	90.30%	100.00%	52.12%	49.70%
7	183	41.60	74.89	0.00%	0.00%	0.00%	0.00%	0.00%	0.00%
8	160	41.63	74.98	100.00%	100.00%	100.00%	100.00%	100.00%	95.15%
9	162	41.15	75.23	100.00%	54.55%	100.00%	66.06%	81.82%	100.00%
10	167	41.64	75.56	100.00%	100.00%	95.15%	44.24%	98.18%	100.00%
11	170	41.63	75.56	54.55%	32.73%	69.09%	13.94%	24.24%	27.88%
12	127	39.62	71.95	100.00%	98.79%	90.30%	100.00%	89.70%	36.97%
13	68	39.63	71.91	100.00%	100.00%	97.58%	73.33%	70.91%	75.15%
14	11	39.60	71.88	100.00%	100.00%	98.18%	100.00%	84.24%	100.00%
15	155	39.43	72.13	11.52%	89.70%	0.00%	0.00%	0.00%	0.00%
16	146	39.32	72.25	15.76%	0.00%	23.03%	0.00%	0.00%	0.00%
17	112	39.37	71.80	100.00%	68.48%	100.00%	50.91%	75.76%	70.91%
18	138	39.32	71.73	100.00%	100.00%	100.00%	100.00%	100.00%	100.00%
19	48	39.70	71.69	100.00%	100.00%	100.00%	100.00%	94.55%	100.00%
20	72	39.70	71.66	99.39%	100.00%	100.00%	81.82%	86.67%	83.64%
21	82	39.69	71.63	100.00%	100.00%	100.00%	100.00%	98.18%	100.00%
22	40	39.48	71.43	100.00%	100.00%	100.00%	100.00%	100.00%	100.00%
23	144	39.32	71.33	100.00%	100.00%	100.00%	75.15%	100.00%	100.00%
24	156	39.36	71.20	100.00%	100.00%	100.00%	74.55%	92.12%	84.85%
25	107	39.03	71.27	100.00%	100.00%	100.00%	100.00%	100.00%	99.39%
26	31	39.05	71.18	100.00%	100.00%	100.00%	100.00%	100.00%	100.00%
27	8	39.03	71.10	100.00%	100.00%	100.00%	100.00%	100.00%	100.00%
28	85	39.26	70.73	100.00%	100.00%	99.39%	100.00%	100.00%	86.06%
29	118	39.35	70.62	100.00%	100.00%	100.00%	100.00%	100.00%	100.00%
30	109	39.24	70.06	100.00%	100.00%	100.00%	100.00%	100.00%	100.00%
31	73	39.22	70.10	100.00%	100.00%	100.00%	100.00%	100.00%	100.00%
32	94	38.63	71.31	99.39%	98.18%	100.00%	100.00%	100.00%	100.00%
33	95	38.63	71.47	83.64%	99.39%	100.00%	100.00%	100.00%	100.00%
34	65	38.65	71.32	100.00%	81.82%	100.00%	100.00%	100.00%	100.00%
35	69	38.68	71.44	100.00%	100.00%	99.39%	100.00%	100.00%	97.58%
36	60	38.68	71.43	100.00%	100.00%	100.00%	100.00%	100.00%	100.00%
37	129	38.69	70.71	92.12%	98.79%	99.39%	84.85%	100.00%	98.18%

FID is the ID of seasonal pumped hydropower storage potential sites in Hunt et al. [6], with IDs 1–11 located in the Syr Darya Basin and IDs 12–37 in the Amu Darya Basin.

REFERENCE:

1. Ma Y., Li Y., Wang H., et al. (2024). Non-deterministic multi-level model for planning water-ecology nexus system under climate change. *ll Rep. Sustain.* **1**:100032. DOI:10.1016/j.crsus.2024.100032
2. Liu S., Luo G. and Wang H. (2020). Temporal and Spatial Changes in Crop Water Use Efficiency in Central Asia from 1960 to 2016. *Sustainability* **12**:572. DOI:10.3390/su12020572
3. Wang X., Zhang J., Wang S., et al. (2023). Reviving the Aral Sea: A Hydro-Eco-Social Perspective. *Earth's Future* **11**:e2023EF003657. DOI:10.1029/2023ef003657
4. Geressu R. T., Siderius C., Rao Kolusu S., et al. (2022). Evaluating the sensitivity of robust water resource interventions to climate change scenarios. *Clim. Risk Manage.* **37**. DOI:10.1016/j.crm.2022.100442
5. Wang F., Huang G. H., Fan Y., et al. (2020). Robust Subsampling ANOVA Methods for Sensitivity Analysis of Water Resource and Environmental Models. *Water Resour. Manage.* **34**:3199-3217. DOI:10.1007/s11269-020-02608-2
6. Hunt J. D., Byers E., Wada Y., et al. (2020). Global resource potential of seasonal pumped hydropower storage for energy and water storage. *Nat. Commun.* **11**:947. DOI:10.1038/s41467-020-14555-y
